# One‐Pot Synthesis of Customized Metal–Phenolic‐Network‐Coated AIE Dots for In Vivo Bioimaging

**DOI:** 10.1002/advs.202104997

**Published:** 2022-02-08

**Authors:** Changhuo Xu, Chen Peng, Xueqin Yang, Ruoyao Zhang, Zheng Zhao, Bo Yan, Jun Zhang, Junyi Gong, Xuewen He, Ryan T. K. Kwok, Jacky W. Y. Lam, Ben Zhong Tang

**Affiliations:** ^1^ Shenzhen Institute of Aggregate Science and Technology School of Science and Engineering The Chinese University of Hong Kong Shenzhen 518172 China; ^2^ Department of Chemistry Hong Kong Branch of Chinese National Engineering Research Center for Tissue Restoration and Reconstruction The Hong Kong University of Science and Technology Hong Kong 999077 China; ^3^ Department of Radiology Shanghai Public Health Clinical Center Fudan University Shanghai 201508 China; ^4^ Center for Aggregation‐Induced Emission SCUT–HKUST Joint Research Institute State Key Laboratory of Luminescent Materials and Devices Guangdong Provincial Key Laboratory of Luminescence from Molecular Aggregates South China University of Technology Guangzhou 510640 China; ^5^ AIE Institute Huangpu Guangzhou 510530 China

**Keywords:** aggregation‐induced emission, coacervation, fluorescence imaging, magnetic resonance imaging, metal–phenolic networks

## Abstract

The integration of aggregation‐induced emission luminogens (AIEgens) and inorganic constituents to generate multifunctional nanocomposites has attracted much attention because it couples the bright aggregate‐state fluorescence of AIEgens with the diverse imaging modalities of inorganic constituents. Herein, a facile and universal strategy to prepare metal–phenolic‐network (MPN)‐coated AIE dots in a high encapsulation efficiency is reported. Through precise control on the nucleation of AIEgens and deposition of MPNs in tetrahydrofuran/water mixtures, termed as coacervation, core–shell MPN‐coated AIE dots with bright emission are assembled in a one‐pot fashion. The optical properties of MPN‐coated AIE dots can be readily tuned by varying the incorporated AIEgens. Different metal ions, such as Fe^3+^, Ti^4+^, Cu^2+^, Ni^2+^, can be introduced to the nanoparticles. The MPN‐coated AIE dots with a red‐emissive AIEgen core are successfully used to perform magnetic resonance/fluorescence dual‐modality imaging in a tumor‐bearing mouse model and blood flow visualization in a zebrafish larva. It is believed that the present study provides a tailor‐made nanoplatform to meet the individual needs of in vivo bioimaging.

## Graphical abstract

A facile strategy is proposed to combine the aggregate‐state emission of aggregation‐induced emission luminogens and the versatility of tannic acid/metal ion complexes within a single nanoparticle via one‐pot synthesis. The obtained nanoparticles are successfully used to perform in vivo dual‐modality imaging and fluorescence‐guided visualization of blood flow in a zebrafish larva.

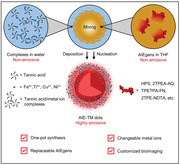

## 1 Introduction

Natural evolution proceeds in a path of balance and holism. By virtue of this principle, natural organic–inorganic (O–I) hybrid materials, such as teeth, bone, shells of marine mussels, reflect the perfect trade‐off properties, including durability, elasticity, toughness, hydrophobicity, and other physicochemical properties.^[^ ^1^ ^]^ To meet the ever‐growing demands of modern manufacturing and living, the applications of artificial O–I composites have been boomingly expanded from ancient straw‐reinforced adobes to advanced composites in the aerospace field because of the development of cross‐cutting synthetic methods toward the precise control on the desired characteristics of O–I hybrids.^[^ ^2^ ^]^ The integration of disparate properties of organic and inorganic constituents via covalent or noncovalent interactions compensates for the inborn defects of a single component and may realize a better performance than parental components due to the unique intercomponent interactions.^[^ ^3^ ^]^ On the other hand, mature nanotechnology lays a solid foundation on the fabrication of nanocomposites with flexible mastery at morphology, size, composition, and interfacial properties, which extends the use of O–I composites in nanooptics,^[^ ^4^ ^]^ nanoelectronics,^[^ ^5^ ^]^ and nanomedicine.^[^ ^6^ ^]^

O–I hybrid nanoparticles are considered as promising candidates for biomedical imaging and therapy in the era of precision medicine.^[^ ^7^ ^]^ In a typical O–I hybrid imaging system, the incorporated inorganic constituents, such as noble metal and iron oxide, allow the implementation of computed tomography and magnetic resonance (MR) imaging to provide anatomic information of interest in high resolution, while the concomitant organic fluorophores enable fluorescence imaging with superior sensitivity.^[^ ^8^ ^]^ Indeed, this synergistic integration enjoys the benefits of fluorescence and other imaging modalities within a single O–I hybrid nanoparticle.^[^ ^9^ ^]^ Fluorescent O–I hybrid nanoparticles can be fabricated through various methods, including surfactant‐based direct wrapping of organic fluorophores and inorganic species, chemical or physical anchoring of organic fluorophores on the surface of inorganic nanoparticles, and other bottom‐up synthetic methods.^[^ ^7^ ^]^ When fluorophores are in close proximity to each other inside the O–I hybrid nanoparticles, their fluorescence is partially or completely quenched due to the detrimental interfluorophore interactions.^[^ ^10^ ^]^ Such a phenomenon of aggregation‐caused quenching (ACQ) is commonly observed in conventional planar fluorophores and can be prevented by minimizing the fluorophore loading content. However, the photostability of the fluorescent O–I hybrid nanoparticles will be significantly sacrificed when the mount of fluorophores is reduced.

Various strategies have been proposed to improve the luminous efficiency of fluorophores in the aggregate state.^[^ ^11^ ^]^ Among them, aggregation‐induced emission (AIE) luminogens elegantly overcome the ACQ problem and its discovery also initiates the thriving study of aggregology.^[^ ^12^ ^]^ The aggregates of AIE luminogens (AIEgens) show brighter emission than their molecular building blocks to enable them as competitive alternatives to construct fluorescent O–I hybrid nanoaggregates.^[^ ^13^ ^]^ The combination of AIEgens and inorganic components to generate O–I hybrids for biomedical applications has been explored, but the present synthetic methods to these composites are still unsatisfactory in terms of universal applicability. For example, the chemical conjugation of AIEgens and metal chelates can unify the originally separated properties of individuals to achieve dual‐modality imaging.^[^ ^14^ ^]^ However, it requires convoluted synthesis and is merely workable for simple and stereotyped tetraphenylethylene (TPE) derivatives with a limited emission wavelength. As may easily be imagined, the development of red‐emissive or near‐infrared‐emissive AIEgen–metal conjugates using chemical synthesis will be extremely laborious and time‐consuming. Thus, it is highly desired to develop a simple yet efficient approach to prepare fluorescent O–I nanocomposites with regulable modules to fulfill the diverse needs of biological applications.

To take this challenge, we herein report a one‐pot synthetic method to fluorescent O–I hybrid nanoparticles with broad applicability. By virtue of a phase separation process, termed as coacervation, nucleation of hydrophobic AIEgens and synergistic deposition of 3D cross‐linked tannic acid/metal ion complexes on the surface of the formed AIEgen nanoaggregates afford core–shell AIEgen/metal ion nanohybrids in tetrahydrofuran (THF)/water mixtures. In this method, both AIEgens and metal ions are replaceable to provide possibilities to customize fluorescent O–I hybrid nanoparticles for extensive theranostic applications. As a proof of concept, in vivo MR and fluorescence imaging of AIEgen/Fe^3+^ nanohybrids are successfully demonstrated.

## 2 Results and Discussion

### 2.1 Synthesis, Characterization, and Photophysical Properties of 2TPEA‐AQ

**Figure 1:**
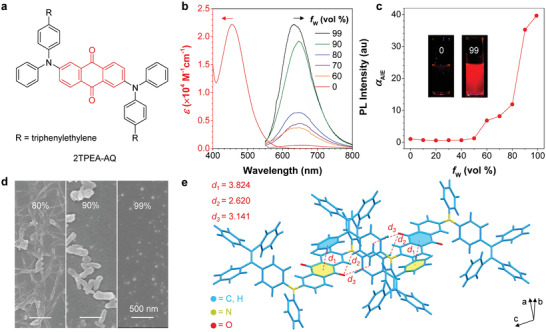
a) Chemical structure of 2TPEA‐AQ. b) Absorption spectrum of 2TPEA‐AQ in THF and its PL spectra in THF/water mixtures with different water fractions (*f*_w_). Concentration: 10 × 10^−6^ m. Excitation wavelength: 470 nm. c) Plot of *α*_AIE_ (*I*/*I*_0_) versus *f*_w_, where *I*_0_ = PL intensity in pure THF. Insets: photos of 2TPEA‐AQ in THF and a THF/water mixture (*f*_w_ = 99%) taken at room temperature under UV illumination. d) SEM images of 2ATPE‐AQ aggregates dispersed in THF/water mixtures with varying water fractions. e) Crystal packing of 2TPEA‐AQ with close contacts.

A new red‐emissive AIEgen, named as 2TPEA‐AQ, was designed and synthesized, as shown in **Figure** **1**a. 2TPEA‐AQ was readily obtained through Buchwald–Hartwig amination using Pd_2_(dba)_3_ and RuPhos as catalyst and ligand, respectively. This strategy is reported to be highly efficient for the formation of C—N bonds.^[^ ^15^ ^]^ Indeed, 2TPEA‐AQ was generated in a high yield of 82%. The resulting molecule was fully characterized by ^1^H and ^13^C NMR spectroscopy, and high‐resolution mass spectrometry with satisfactory results (Figures S1–S3, Supporting Information). The photophysical properties of 2TPEA‐AQ were studied by UV–vis and photoluminescence (PL) spectroscopy. As shown in Figure 1b, 2TPEA‐AQ gives an absorption maximum at 455 nm. Electron and hole natural transition orbital analysis in the gas phase suggested that the low‐energy absorption band of the molecule was contributed by highest occupied molecular orbital (HOMO) → lowest unoccupied molecular orbital (LUMO) and HOMO‐2 → LUMO transitions with coefficients of 0.599 and 0.231, respectively (Figure S4, Supporting Information). The AIE effect of 2TPEA‐AQ was studied in THF/water mixtures with varying water fractions. In the pure THF solution, 2TPEA‐AQ barely fluoresced (Figure 1b,c). It indicates that the motorized molecular structure of 2TPEA‐AQ facilitates the nonradiative decay in the solution state. Even up to 50% of water was added to the THF solution, no obvious fluorescence was detected. However, when the water fraction reached 60%, the intrinsic emission of 2TPEA‐AQ started to recover and reached its maximum intensity at a water fraction of 99% (Figure 1c). Such a photophysical behavior demonstrates the AIE characteristic of 2TPEA‐AQ. The PL spectrum of 2TPEA‐AQ powders was peaked at 630 nm and the associated PL quantum yield was determined to be 5.9% by an integrating sphere. Additionally, the fluorescence lifetime was calculated to be 2.5 ns (Figure S5b, Supporting Information). This result suggests that 2TPEA‐AQ emits no delayed fluorescence, which is different from the reported anthraquinone‐based compounds.^[^ ^16^ ^]^

Scanning electron microscope (SEM) was then utilized to investigate the morphology of 2TPEA‐AQ aggregates formed in different THF/water fractions (Figure 1d). Distorted nanofibers were observed at 80% water fraction. Further increasing the water fraction to 90% led to the formation of rod‐like particles. When the THF content was minimized to 1%, the 2TPEA‐AQ molecules were conformably clustered into nanoparticles. Thus, the size of 2TPEA‐AQ aggregates became smaller at increasing water content. This intriguing aggregation behavior of 2TPEA‐AQ inspired us to probe its real molecular conformation and arrangement by single‐crystal analysis. Single crystals of 2TPEA‐AQ were grown in vials at ambient conditions by slow diffusion of hexane into its chloroform solution. As depicted in Figure 1e and Table S1 (Supporting Information), 2TPEA‐AQ crystallizes in the triclinic *P*−1 space group with an elemental cell containing two molecules. Its crystal packing shows various sorts of intermolecular interactions, including C—H···O and partial *π*···*π* interactions in the crystal lattice. On this occasion, the intramolecular motions of 2TPEA‐AQ are locked due to the physical constraints, which retards the nonradiative relaxation pathways to allow 2TPEA‐AQ to fluoresce intensively. Unfortunately, after standing for some time, these emissive nanoaggregates tended to agglomerate into visible particles, suggestive of the colloidal instability of 2TPEA‐AQ nanoaggregates in water. Such a drawback limits their applications in biological environment.

### 2.2 Preparation and Characterization of 2TPEA‐AQ@AIE‐TFe Dots

**Figure 2:**
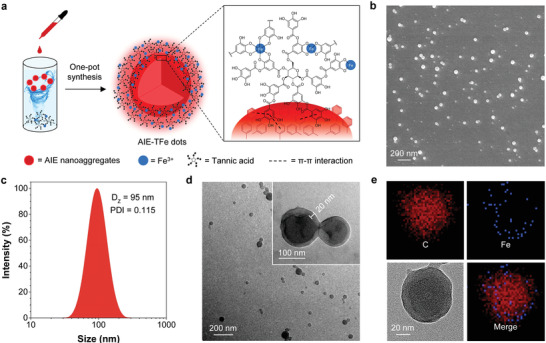
a) Schematic illustration of one‐pot synthesis of AIE‐TFe dots in a THF/water mixture (*f*_w_ = 80%). [AIEgen] = [Fe^3+^] = [TA] = 0.2 × 10^−3^ m. b) SEM image of 2TPEA‐AQ@AIE‐TFe dots. c) DLS size distribution of 2TPEA‐AQ@AIE‐TFe dots. d) TEM images of 2TPEA‐AQ@AIE‐TFe dots. e) Element mapping images of carbon (C) and iron (Fe) throughout 2TPEA‐AQ@AIE‐TFe dots by energy dispersive spectroscopy.

Tannic acid (TA) is a biocompatible and water‐soluble compound approved by the US Food and Drug Administration. As a typical plant polyphenol, TA can serve as a templating and stabilizing agent in the preparation of mesoporous silica nanoparticles, gold nanoparticles, and silver nanoparticles.^[^ ^17^ ^]^ Furthermore, the coordination of TA and metal ions can yield adhesive metal–phenolic networks (MPNs).^[^ ^18^ ^]^ The deposition of O–I hybrid MPNs on multifarious nanomaterials can endow them with an excellent colloidal stability and advanced biological applications.^[^ ^19^ ^]^ Unlike traditional hydrophilic dyes, AIEgen nanoaggregates can provide an idea template for the coating of MPNs due to their hydrophobic nature. Therefore, it deserves to explore the possibility of interfacial adhesion on AIEgen nanoaggregates using MPNs to construct fluorescent O–I hybrid AIE dots. TA/Fe^3+^ complexes were used to test our hypothesis. As shown in **Figure** **2**a, a THF solution of 2TPEA‐AQ was dropwise added into an aqueous solution of TA/Fe^3+^ complexes under rapid stirring, where the three solutes were kept at an identical molar concentration, followed by raising the pH of the resultant mixture using a 3‐(N‐morpholino)propanesulfonic acid (MOPS) buffer. Surprisingly, scattered colloidal nanoparticles, coined as 2TPEA‐AQ@AIE‐TFe dots, were observed by SEM (Figure 2b). Dynamic light scattering (DLS) analysis of 2TPEA‐AQ@AIE‐TFe dots showed an average size of 95 nm with a polydispersity index (PDI) of 0.115 (Figure 2c and Table S3 (Supporting Information)). As comparison, AIE dots wrapped by 1, 2‐Distearoyl‐sn‐glycero‐3‐phosphoethanolamine‐Poly(ethylene glycol) (DSPE‐PEG) were fabricated as a control with a PDI of 0.233 (Figures S17 and S21a, Supporting Information). Obviously, the new nanofabrication method can provide a respectable PDI for the practical application of 2TPEA‐AQ@AIE‐TFe dots.

The successful preparation of 2TPEA‐AQ@AIE‐TFe dots motivates us to thoroughly investigate the entire self‐assembly procedure. The morphology of TA/Fe^3+^ complexes before the addition of 2TPEA‐AQ was characterized by transmission electron microscopy (TEM). Figure S6 (Supporting Information) reveals that TA and Fe^3+^ in water with a feed mole ratio of 1:1 could form ununiform nanocomplexes with a diameter of ≈10 nm. The addition of 2TPEA‐AQ to the abovementioned solution resulted in the formation of core–shell nanoparticles, as evidenced by the TEM images in Figure 2d and Figure S7 (Supporting Information). To validate the core–shell structure, 2TPEA‐AQ@AIE‐TFe dots residing on a copper grid were subjected to the treatment of THF etching several times, as THF dissolved only AIEgens rather than TA/Fe^3+^ complexes. After the removal of AIEgens, hollow structure was observed by TEM in Figure S8 (Supporting Information). It indicates that the core part is exactly the nanoaggregates of 2TPEA‐AQ. Element mapping was performed for 2TPEA‐AQ@AIE‐TFe dots by energy dispersive spectroscopy. As shown in Figure 2e, the blue dots representing iron elements scatter on the peripheral region of the nanoparticles, which suggests that TA/Fe^3+^ complexes compose the shell with high contrast. The energy‐dispersive X‐ray spectrum (EDX) and quantitative results of elemental compositions (C, N, O, and Fe) of 2TPEA‐AQ@AIE‐TFe dots are shown in Figure S9 and Table S2 (Supporting Information). The atomic content of the doped iron was measured to be 0.26% and the surface zeta potential of the core–shell nanoparticles was determined to be −28.3 mV probably due to the deprotonation of TA molecules on the surface of the nanoparticles (Table S3, Supporting Information). X‐ray photoelectron spectroscopy (XPS) provides the composition information of the extreme surface area (1–10 nm) of the investigated materials. As shown in Figure S10a (Supporting Information), both the measured oxygen and iron contents by XPS are significantly higher than the EDX results. Therefore, it testifies the presence of TA and iron in the outer shell of 2TPEA‐AQ@AIE‐TFe dots. Figure S10b (Supporting Information) reveals a Fe 2p_3/2_ peak at 712.6 eV with a split of 2p peak of 13 eV, indicating the existence of Fe^3+^ in the TA/Fe^3+^ complexes. The O 1s core‐level spectrum in Figure S10c (Supporting Information) displays the peaks at 533.7 and 532.3 eV. The peak at 533.7 eV could be assigned to HO—C groups of TA and HO—Fe species, while another peak at 532.3 eV could be attributed to O═C of TA and O—Fe species, respectively.^[^ ^20^ ^]^

### 2.3 Coacervation and Universal Applicability

**Figure 3:**
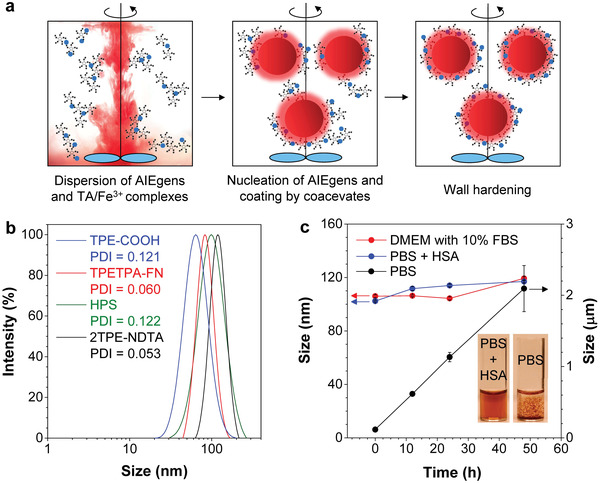
a) Pictorial representation of the coacervation process involving i) dispersion of AIEgens and TA/Fe^3+^ complexes, ii) nucleation of AIEgens and deposition of coacervates, and iii) wall hardening. b) Size distributions of AIE‐TFe dots in water prepared using different AIEgens. [AIEgen] = [Fe^3+^] = [TA] = 0.2 × 10^−3^ m. c) Stability of 2TPEA‐AQ@AIE‐TFe dots in Dulbecco's Modified Eagle Medium (DMEM) containing 10% fetal bovine serum, phosphate buffer solution (PBS, pH = 7.4), and PBS supplemented with human serum albumin (HSA). Insets: solutions of 2TPEA‐AQ@AIE‐TFe dots in the presence or absence of HSA after incubation at room temperature for 48 h.

To further decipher the self‐assembly mechanism of TA/Fe^3+^ complexes and AIEgen aggregates in the THF/water mixture, a concept, termed as coacervation or phase separation, is introduced.^[^ ^21^ ^]^ Coacervation describes a process in which the precipitation of polymer aggregates (coacervates) on the surface of minute core materials is triggered by external stimuli, including temperature, pH, antisolvent, salt, etc.^[^ ^22^ ^]^ Likewise, the self‐assembly process in our case is a typical example of coacervation. As shown in **Figure** **3**a, the preparation of the nanoparticles begins with the dispersion of AIEgens and TA/Fe^3+^ complexes in THF/water mixtures. The nucleation of AIEgens rapidly develops once the THF solution becomes supersaturated upon mixing with water. At this very moment, the sufficient mixing of THF and water also squeezes the previously dissolved TA/Fe^3+^ complexes from the solution to form a coacervate phase. As evidenced in Figure S11 (Supporting Information), the desolvation of TA/Fe^3+^ complexes caused by the addition of THF resulted in the generation of massive micrometer‐sized aggregates. Thus, THF was thought to serve as a coacervation agent or an inducing agent. Subsequently, the deposition of TA/Fe^3+^ complexes on AIEgen aggregates occurs owing to the strong *π*–*π* interactions between the phenyl rings of AIEgens and the galloyl groups of TA (Figure 2a).^[^ ^23^ ^]^ At last, the adjustment of pH to neutral allows the completion of wall hardening to afford TA‐/Fe^3+^‐complex‐coated AIE dots. It is noteworthy that maintaining the nucleation rate of AIEgens and the deposition rate of TA/Fe^3+^ complexes in balance is vitally important in the self‐assembly procedure, otherwise big aggregates or insoluble precipitates may form.

To certify the universal applicability of the proposed strategy, other AIEgens, such as Hexaphenylsilole (HPS) and TPETPA‐FN, were used to fabricate AIE‐TFe dots. Both AIEgens could be shaped into colloidal nanoparticles upon self‐assembly with TA/Fe^3+^ complexes (Figure 3b and Figure S12 (Supporting Information)). 2TPE–NDTA is a reported photothermal agent that harvests and transfers photoenergy as heat,^[^ ^24^ ^]^ and it was fabricated into AIE‐TFe dots using the same method (Figure S12c, Supporting Information). The obtained nanoparticles can potentially be applied in photothermal therapy and photoacoustic imaging with the given functionalities of TA/Fe^3+^ complexes. The PDI of TPETPA‐FN@AIE‐TFe dots, HPS@AIE‐TFe dots, and 2TPE–NDTA@AIE‐TFe dots is 0.06, 0.122, and 0.053, respectively. Therefore, the uniformity of AIE dots fabricated using the one‐pot method is decent. Besides uncharged AIEgens, TPE‐COOH is a negatively charged AIEgen and formed vesicles with TA/Fe^3+^ complexes due to the additional coordination between the carboxylic acid group and Fe^3+^ (Figure S13, Supporting Information). However, the present strategy is not applicable to AIEgens with a positive charge, such as TPE–TMX and TPE–EP (Figure S14, Supporting Information). Macroscopic aggregates were formed when TPE–TMX or TPE–EP was mixed with TA/Fe^3+^ complexes. The formation of large aggregates may be ascribed to the low surface zeta potential of AIE‐TFe dots resulting from the neutralization effect of the positively charged AIEgens in the inner part, which provokes the agglomeration of AIE‐TFe dots.

The stability of 2TPEA‐AQ@AIE‐TFe dots was investigated in a phosphate buffer solution (PBS) and a commonly used cell culture medium, namely, Dulbecco's Modified Eagle Medium (DMEM), with 10% fetal bovine serum (FBS). As shown in Figure 3c, the size of 2TPEA‐AQ@AIE‐TFe dots in PBS increases with increasing incubation time, which reveals that AIE‐TFe dots tend to agglomerate into microparticles in a concentrated salt solution due to the charge screening effect of salts.^[^ ^25^ ^]^ However, the nanoparticles exhibit high stability in DMEM. Given that TA can form multiple hydrogen bonds and hydrophobic interactions with proteins,^[^ ^26^ ^]^ it is inferred that proteins may work as stabilizers since there are plenty of proteins in DMEM with 10% FBS. Thus, the stability testing assay was carried out by incubation of human serum albumin (HSA) and 2TPEA‐AQ@AIE‐TFe dots in PBS. The result in Figure 3c suggests that the addition of HSA indeed avoids the agglomeration of nanoparticles and reinforces their stability in PBS. This discovery demonstrates the great potential of AIE‐TFe dots for bioapplications since proteins always exist in the biological environment.

### 2.4 Tunable Features of 2TPEA‐AQ@AIE‐TM Dots

**Figure 4:**
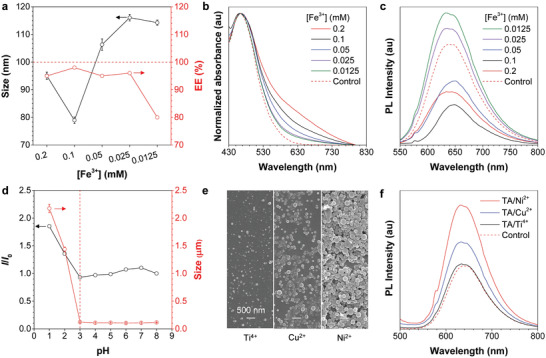
a) Effect of Fe^3+^ concentration on the size and encapsulation efficiency (EE) of 2TPEA‐AQ@AIE‐TFe dots while the concentrations of 2TPEA‐AQ and TA were fixed at 0.2 × 10^−3^ m. b) Absorption and c) PL spectra of 2TPEA‐AQ@AIE‐TFe dots in water prepared at varying Fe^3+^ concentrations; AIE dots fabricated using DSPE–PEG as a surfactant were used as a control group; the concentration of 2TPEA‐AQ in all prepared AIE dots was 0.2 × 10^−3^ m. d) Relative PL intensity (*I*/*I*_0_) and size change of 2TPEA‐AQ@AIE‐TFe dots at different pH after incubation in the corresponding solutions for 24 h, where *I*_0_ = PL intensity of 2TPEA‐AQ@AIE‐TFe dots at pH = 8. e) SEM images and f) PL spectra of 2TPEA‐AQ@AIE‐TM dots (M = Ti, Cu, or Ni) prepared with different metal ions (M*^n^* ^+^ = Ti^4+^, Cu^2+^, or Ni^2+^). [M*^n^* ^+^] = [2ATPE‐AQ] = [TA] = 0.2 × 10^−3^ m.

The effect of Fe^3+^ concentration on the size and encapsulation efficiency (EE) of 2TPEA‐AQ@AIE‐TFe dots was investigated by DLS and SEM. Both 2TPEA‐AQ and TA concentrations were kept constant at 0.2 × 10^−3^ m. The EE of 2TPEA‐AQ@AIE‐TFe dots was calculated using UV–vis spectrophotometry (Figure S15 and Table S3, Supporting Information). When the concentration of Fe^3+^ was varied from 0.2 × 10^−3^ to 0.0125 × 10^−3^ m, the EE remained at above 80%, indicative of the high efficiency of the wrapping method (**Figure** **4**a). The size of 2TPEA‐AQ@AIE‐TFe dots initially decreased slightly and then increased with the increased Fe^3+^ concentration. The SEM images given in Figure S16 (Supporting Information) demonstrate spherical AIE‐TFe dots irrespective of the Fe^3+^ concentration. However, in the absence of Fe^3+^, no nanoparticles were formed because TA is too hydrophilic to encapsulate 2TPEA‐AQ nanoaggregates. When the Fe^3+^ concentration was raised to 0.4 × 10^−3^ m, the resulting nanoparticles were rapidly precipitated. Therefore, the successful coacervation of AIE‐TFe dots requires a moderate ratio of TA to Fe^3+^ to warrant the stability of formed AIE‐TFe dots.

The photophysical properties of 2TPEA‐AQ@AIE‐TFe dots with varying Fe^3+^ concentrations were characterized by UV–vis and PL spectroscopy (Figure 4b,c). As shown in Figure 4b, the absorption in the near‐infrared (NIR) region is gradually elevated by increasing the Fe^3+^ concentration. Meanwhile, the absorption spectrum of TA/Fe^3+^ complexes in water displays a peak at 580 nm, which is assigned to the ligand‐to‐metal charge‐transfer band (Figure S20a, Supporting Information).^[^ ^27^ ^]^ Thus, the increased absorption of 2TPEA‐AQ@AIE‐TFe dots is ascribed to the coordination of TA and Fe^3+^. Figure 4c shows that 2TPEA‐AQ@AIE‐TFe dots become more emissive upon decreasing the Fe^3+^ concentration. At a Fe^3+^ concentration of 0.025 × 10^−3^ or 0.0125 × 10^−3^ m, the nanoparticles show stronger emission than the DSPE–PEG‐wrapped AIE dots. It manifests that the fluorescence of 2TPEA‐AQ@AIE‐TFe dots can be regulated via dark resonance energy transfer (DRET) from fluorescent AIEgens to nonfluorescent TA/Fe^3+^ complexes.^[^ ^28^ ^]^ The 2TPEA‐AQ@AIE‐TFe dots prepared at a Fe^3+^ concentration of 0.1 × 10^−3^ m exhibited an abnormally low PL owing to their small size (Figure 4a). It enlarges the specific surface area to facilitate the excited‐state molecular motion of the AIEgens and the DRET process to quench the emission.^[^ ^29^ ^]^

The molar ratio of three components in AIE‐TFe dots was optimized to be 1:1:1 for the following investigations. The coordination behavior of TA and Fe^3+^ was reported to be pH‐dependent,^[^ ^30^ ^]^ and there are three transition states of TA/Fe^3+^ complexes at different pH ranges, including mono‐, bis‐, and tris‐complex states (Scheme S1, Supporting Information). Thus, the PL and size change of 2TPEA‐AQ@AIE‐TFe dots at different pH were investigated (Figure 4d). By a gradual decrease of pH from 8 to 1, both the PL and size kept almost unchanged in the initial stage. Subsequently, an abrupt change point was identified at pH ≈ 3, where a transition from the bis‐complex state to the mono‐complex state may happen on the surface of the nanoparticles, as proved by the absorption decrease in Figure S18b (Supporting Information). Definitely, the disassembly of TA/Fe^3+^ complexes reduces the DRET efficiency to intensify the fluorescence (Figure S18a, Supporting Information). Meanwhile, the destructed shell part at pH < 3 caused the agglomeration of the nanoparticles (Figure S18c,d, Supporting Information). On the other hand, the external galloyl groups were protonated at such a low pH to neutralize the surface zeta potential, which also induced the growth of the visible aggregates. The release of Fe^3+^ from 2TPEA‐AQ@AIE‐TFe dots upon dialysis was studied in PBS at pH = 5.0 and pH = 7.4 (Figure S19, Supporting Information). Results suggested that incubation of the nanoparticles in the acidic environment truly accelerated the release of Fe^3+^ and boosted the fluorescence.

To enrich the scope of the incorporated metal ions, other metal ions, including titanium cations (Ti^4+^), copper cations (Cu^2+^), and nickel cation (Ni^2+^), were utilized to prepare nanoparticles namely 2TPEA‐AQ@AIE‐TTi dots, 2TPEA‐AQ@AIE‐TCu dots, and 2TPEA‐AQ@AIE‐TNi dots, respectively. The coordination of TA and Ti^4+^ in water gave a yellow solution with an absorption band close to 400 nm (Figure S20b, Supporting Information), while the aqueous solutions of TA/Cu^2+^ and TA/Ni^2+^ complexes were colorless observed by naked eyes (Figure S20c,d, Supporting Information). As shown in Figure 4e, three types of metal ions can self‐assemble with TA and 2TPEA‐AQ to afford stable spherical nanoparticles. The DLS data shown in Figure S21a (Supporting Information) suggest that the size of 2TPEA‐AQ@AIE‐TTi dots is smaller than those of 2TPEA‐AQ@AIE‐TCu dots and 2TPEA‐AQ@AIE‐TNi dots, which is consistent with the gradually increased absorption in the NIR region owing to the scattering effect of the large particles (Figure S21b, Supporting Information). It is worthy to note that both Fe^3+^ and Ti^4+^ can be regarded as hard Lewis acids, and they can bind to TA strongly. By contrast, both Cu^2+^ and Ni^2+^ are moderate Lewis acids and they show relatively small binding constants with TA.^[^ ^31^ ^]^ Therefore, the fast deposition rate of TA/Ti^4+^ complexes due to the rapid 3D cross‐linking of TA/Ti^4+^ complexes prevents the nucleation of 2TPEA‐AQ to some extent to generate small‐sized 2TPEA‐AQ@AIE‐TTi dots. XPS results verify the existence of metal ions in the corresponding nanoparticles (Figure S21c–f and Table S4, Supporting Information). Figure 4f manifests that 2TPEA‐AQ@AIE‐TCu dots and 2TPEA‐AQ@AIE‐TNi dots emit stronger PL than DSPE–PEG‐wrapped AIE dots and 2TPEA‐AQ@AIE‐TTi dots. In short, the size and fluorescence of AIE‐TM (M = Fe, Ti, Cu, Ni) dots are modulable. By employing variable MPNs as the shell and replaceable AIEgens as the core, the obtained nanoparticles can be tailor‐made in terms of specific requirements for practical applications.

### 2.5 Dual‐Modality Imaging of 2TPEA‐AQ@AIE‐TFe Dots

**Figure 5:**
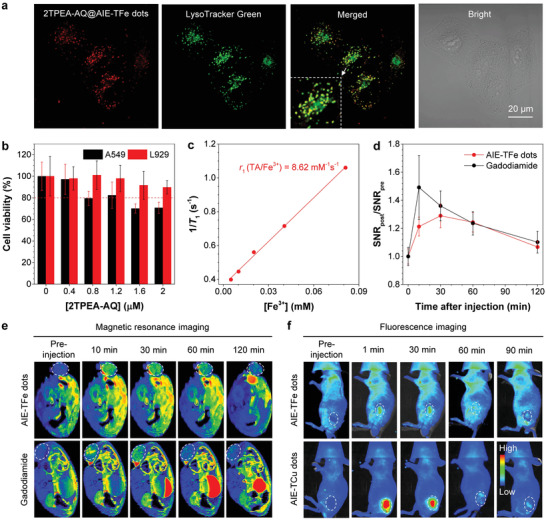
a) Confocal images of A549 cells costained with 2TPEA‐AQ@AIE‐TFe dots and LysoTracker Green. The excitation wavelength and the wavelength range of the emission filter were 488 and 650–700 nm for 2TPEA‐AQ@AIE‐TFe dots, and 488 and 500–550 nm for LysoTracker Green, respectively. b) Viability of A549 cells treated with different concentrations of 2TPEA‐AQ@AIE‐TFe dots after 24 h incubation. c) Linear fitting of 1/*T*_1_ of 2TPEA‐AQ@AIE‐TFe dots in water as a function of Fe^3+^ concentration. d) Change in the MR signal‐to‐noise ratio (SNR) in the tumor region over time after intratumoral injection of 2TPEA‐AQ@AIE‐TFe dots or gadodiamide into A549 tumor‐bearing mice. The molar concentration of Fe^3+^ in 2TPEA‐AQ@AIE‐TFe dots was equal to that of gadodiamide. e) In vivo *T*_1_‐weighted MR transverse pseudocolor images of A549 tumor‐bearing mouse at different time points. The dashed line of the ellipse indicates the tumor region. f) In vivo fluorescence images of A549 tumor‐bearing mouse at different time points after intratumoral injection of 2TPEA‐AQ@AIE‐TFe dots or 2TPEA‐AQ@AIE‐TCu dots.

AIE‐TM dots potentially provide a versatile nanoplatform for multimodality imaging because of the integration of advantages of MPNs and AIEgens. As a demonstration, 2TPEA‐AQ@AIE‐TFe dots were first used as visualizers for in vitro and in vivo fluorescence imaging. A549 lung cancer cells were costained with 2TPEA‐AQ@AIE‐TFe dots and LysoTracker Green and the results were shown in **Figure** **5**a. After incubation with cells for 5 h, most 2TPEA‐AQ@AIE‐TFe dots resided in endo‐/lysosomes, as reflected by the yellow dots in the merged image. The acidic microenvironment in endo‐/lysosomes (pH = 4.5–5) is speculated to trigger the agglomeration of 2TPEA‐AQ@AIE‐TFe dots after internalization by endocytosis. The agglomerates of the nanoparticles fail to escape from the endo‐/lysosomes and are trapped there. In vitro cytotoxicity of 2TPEA‐AQ@AIE‐TFe dots to fibroblast L929 cells of normal mouse and A549 cells was evaluated by Cell Counting Kit‐8. While 2TPEA‐AQ@AIE‐TFe dots exhibited negligible cytotoxicity to L929 cells, the A549 cells retained a high viability of over 80% in the presence of low concentration of 2TPEA‐AQ@AIE‐TFe dots (Figure 5b). When the concentration of the nanoparticles was raised to 1.6 × 10^−6^ or 2 × 10^−6^ m, slight toxicity was observed. The higher uptake and retention of the nanoparticles in cancer cells than normal cells and the innate anticancer activity of TA may lead to the slight cellular toxicity.^[^ ^32^ ^]^

To demonstrate 2TPEA‐AQ@AIE‐TFe dots as an effective MR contrast agent, their spin–lattice relaxation time or a time constant known as *T*_1_, was measured in the aqueous solution as a function of Fe^3+^ concentration. The longitudinal relaxivity (*r*_1_) of 2TPEA‐AQ@AIE‐TFe dots was determined to be around 8.62 mm ^−1^ s^−1^, which is higher than those of the reported TA‐/Fe^3+^‐complex‐coated nanoparticles (Figure 5c).^[^ ^33^ ^]^ The high relaxivity and red fluorescence of 2TPEA‐AQ@AIE‐TFe dots enabled their further application for in vivo dual‐modality imaging. 2TPEA‐AQ@AIE‐TFe dots were intratumorally injected into A549 tumor‐bearing mice. Gadodiamide is a marketed gadolinium‐based contrast agent, and was also injected into the same animal model as a control. *T*_1_‐weighted MR transverse pseudocolor images of A549 tumor‐bearing mice and MR signal‐to‐noise ratio of the tumor region were obtained at different time points (Figure 5d,e). Compared with gadodiamide, 2TPEA‐AQ@AIE‐TFe dots showed a nearly equal contrast ability and their corresponding signal lasted a bit longer in the tumor region due to the diffusion‐resistance nature of the nanoparticles. The fluorescence imaging performance of 2TPEA‐AQ@AIE‐TFe dots is shown in Figure 5f. The fluorescence signal vanished with time, which agreed well with the results of MR imaging. 2TPEA‐AQ@AIE‐TCu dots showed brighter emission in the tumor site after intratumoral injection. This demonstrates that the imaging ability of AIE‐TM dots can be readily regulated to meet the specific needs in bioimaging. The Hematoxylin and Eosin (H&E) staining images of tumor sections at different time points indicated the good biosafety of 2TPEA‐AQ@AIE‐TFe dots (Figure S22a, Supporting Information). 2TPEA‐AQ@AIE‐TFe dots were then injected intravenously into the A549 tumor model to observe the enhanced permeability and retention effect.^[^ ^34^ ^]^ As revealed in Figure S22b,c (Supporting Information), the MR signal of the nanoparticles was first intensified slowly and then declined after reaching the maximum. To qualitatively assess the biodistribution of 2TPEA‐AQ@AIE‐TFe dots after intravenous injection, the main organs, such as heart, liver, spleen, lung, kidney, and tumor of the mice were sectioned and their fluorescence images were acquired (Figure S23, Supporting Information). Most 2TPEA‐AQ@AIE‐TFe dots were found to accumulate in the liver and spleen. All animal experiments were performed in accordance with the guidelines of the ethical committee of Shanghai Public Health Clinical Center and the regulations of the National Ministry of Health.

### 2.6 Visualization of Blood Flow in a Zebrafish Larva

**Figure 6:**
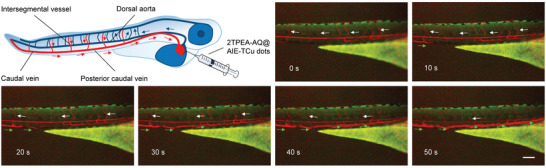
Fluorescent images of a wild‐type zebrafish larva (zebrafish strain: AB) at different time points after injection of 2TPEA‐AQ@AIE‐TCu dots into its heart. Scale bar = 100 µm; excitation wavelength: 485 nm.

The blood vascular network in zebrafish larvae is a circuit model composed of dorsal aorta, segmental vessels, cardinal vein, and posterior cardinal vein (**Figure** **6**).^[^ ^35^ ^]^ In this model, blood is generally pumped out of the heart and exits the dorsal aorta. At the end of the tail, the blood flow will turn 180° and exit the cardinal vein as well as posterior cardinal vein. Therefore, the dorsal aorta, cardinal vein, and posterior cardinal vein were chosen as regions of interest. Based on the bright fluorescence of 2TPEA‐AQ@AIE‐TCu dots, they were selected to inject into the heart of a wild‐type zebrafish larva (zebrafish strain: AB) at 2 days postfertilization (Figure S24, Supporting Information). The living zebrafish larva was then imaged under a fluorescence microscope. As shown in Figure 6 and Movie S1 (Supporting Information), the strong red fluorescence enables the clear observation of the dorsal aorta at 0 s. The fluorescence in the dorsal aorta gradually became weaker with time. After 10 s, the blood carrying the nanoparticles started to enter the posterior cardinal vein. At last, all the injected nanoparticles, which traveled throughout the vascular network, converged in the vein to emit strong fluorescence. It is believed that the negative zeta potential of the TA‐/Cu^2+^‐complex‐coated nanoparticles minimizes the interactions with the blood vascular wall and contributes to the prolonged retention time in the bloodstream. As a control experiment shown in Figure S25 (Supporting Information), 2TPEA‐AQ nanoaggregates were directly injected into the heart of a zebrafish larva, and no red emission was observed in the blood flow postinjection. All the nanoaggregates were rapidly precipitated and the red emission was only found at the injection point. The result manifests that using TA/Cu^2+^ complexes as the coatings of AIEgen nanoaggregates can prevent the biofouling of AIEgen nanoaggregates and improve the colloidal stability of AIE dots when they circulate through the vasculature. Therefore, 2TPEA‐AQ@AIE‐TCu dots give a striking fluorescence imaging performance of the whole‐body vascular system in a dynamic and real‐time way.

## 3 Conclusion

In this work, we have provided a highly effective strategy to couple AIEgens with MPNs to form core–shell fluorescent O–I nanohybrids. 2TPEA‐AQ@AIE‐TFe dots were constructed via facile coacervation and displayed excellent colloidal stability in the protein‐rich biological environment. Their size and fluorescence could be readily regulated. The AIEgens and metal ions were replaceable to provide access to tailor‐made AIE‐TM dots. As a proof‐of‐concept study, 2TPEA‐AQ@AIE‐TFe dots were successfully used to perform in vivo dual‐modality imaging. Fluorescence‐guided visualization of blood flow was performed by 2TPEA‐AQ@AIE‐TCu in a zebrafish larva. The proposed strategy delicately resolves the outstanding conundrum of the flexible integration of AIEgens and inorganic constituents and greatly promotes the development of AIEgen‐incorporated O–I nanocomposites with expected features. As the aggregation of AIEgens occurs in step with the deposition of TA/Fe^3+^ complexes, the present two‐in‐one method enables the continuous and scalable production of MPN‐coated AIE dots, which is of guiding significance for practical nanofabrication.

## Conflict of Interest

The authors declare no conflict of interest.

## Supporting information

Supporting Information

Supplemental Movie 1

## Data Availability

The data that support the findings of this study are available from the corresponding author upon reasonable request.

## References

[1] C. Sanchez , P. Belleville , M. Popall , L. Nicole , Chem. Soc. Rev. 2011, 40, 696. 21229132 10.1039/c0cs00136h

[2] P. Gomez‐Romero , Adv. Mater. 2001, 13, 163.

[3] F. Mammeri , E. L. Bourhis , L. Rozes , C. Sanchez , J. Mater. Chem. 2005, 15, 3787.

[4] P. Innocenzi , B. Lebeau , J. Mater. Chem. 2005, 15, 3821.

[5] J. A. Chang , S. H. Im , Y. H. Lee , H.‐j. Kim , C.‐S. Lim , J. H. Heo , S. I. Seok , Nano Lett. 2012, 12, 1863. 22401668 10.1021/nl204224v

[6] Y. Liu , Y. Zhao , X. Chen , Theranostics 2019, 9, 3122. 31244945 10.7150/thno.31918 PMC6567971

[7] N. Zhao , L. Yan , X. Zhao , X. Chen , A. Li , D. Zheng , X. Zhou , X. Dai , F.‐J. Xu , Chem. Rev. 2018, 119, 1666. 30592420 10.1021/acs.chemrev.8b00401

[8] P. J. Cassidy , G. K. Radda , J. R. Soc., Interface 2005, 2, 133. 16849174 10.1098/rsif.2005.0040 PMC1629073

[9] S. Lee , X. Chen , Mol. Imaging 2009, 8, 87. 19397854

[10] T. Förster , K. Kasper , Z. Elektrochem., Ber. Bunsen‐Ges. 1955, 59, 976.

[11] a) L. Yao , S. Zhang , R. Wang , W. Li , F. Shen , B. Yang , Y. Ma , Angew. Chem., Int. Ed. 2014, 53, 2119; 10.1002/anie.201308486 24453193

[12] a) Z. Zhao , H. Zhang , J. W. Lam , B. Z. Tang , Angew. Chem., Int. Ed. 2020, 59, 9888; 10.1002/anie.201916729 32048428

[13] a) J. Huang , B. He , Z. Zhang , Y. Li , M. Kang , Y. Wang , K. Li , D. Wang , B. Z. Tang , Adv. Mater. 2020, 32, 2003382; 10.1002/adma.202003382 32761671

[14] a) Y. Chen , M. Li , Y. Hong , J. W. Lam , Q. Zheng , B. Z. Tang , ACS Appl. Mater. Interfaces 2014, 6, 10783; 24942209 10.1021/am502282f

[15] W. W. Lee , Z. Zhao , Y. Cai , Z. Xu , Y. Yu , Y. Xiong , R. T. Kwok , Y. Chen , N. L. Leung , D. Ma , J. W. Y. Lam , A. Qin , B. Z. Tang , Chem. Sci. 2018, 9, 6118. 30210763 10.1039/c8sc01377b PMC6118221

[16] Q. Zhang , H. Kuwabara , W. J. Potscavage Jr , S. Huang , Y. Hatae , T. Shibata , C. Adachi , J. Am. Chem. Soc. 2014, 136, 18070. 25469624 10.1021/ja510144h

[17] a) T. Ahmad , J. Nanotechnol. 2014, 2014, 954206;

[18] a) M. J. Harrington , A. Masic , N. Holten‐Andersen , J. H. Waite , P. Fratzl , Science 2010, 328, 216; 20203014 10.1126/science.1181044 PMC3087814

[19] a) W. Xie , Z. Guo , L. Zhao , Y. Wei , Theranostics 2021, 11, 6407; 33995665 10.7150/thno.58711 PMC8120219

[20] a) C. Maerten , L. Lopez , P. Lupattelli , G. Rydzek , S. Pronkin , P. Schaaf , L. Jierry , F. Boulmedais , Chem. Mater. 2017, 29, 9668;

[21] Y. P. Timilsena , T. O. Akanbi , N. Khalid , B. Adhikari , C. J. Barrow , Int. J. Biol. Macromol. 2019, 121, 1276. 30352231 10.1016/j.ijbiomac.2018.10.144

[22] a) A. Trojanowska , A. Nogalska , R. G. Valls , M. Giamberini , B. Tylkowski , Phys. Sci. Rev. 2017, 2, 20170020;

[23] D. Lin , B. Xing , Environ. Sci. Technol. 2008, 42, 5917. 18767645 10.1021/es800329c

[24] Z. Zhao , C. Chen , W. Wu , F. Wang , L. Du , X. Zhang , Y. Xiong , X. He , Y. Cai , R. T. K. Kwok , J. W. Y. Lam , X. Gao , P. Sun , D. L. Phillips , D. Ding , B. Z. Tang , Nat. Commun. 2019, 10, 768. 30770816 10.1038/s41467-019-08722-z PMC6377612

[25] T. Park , W. I. Kim , B. J. Kim , H. Lee , I. S. Choi , J. H. Park , W. K. Cho , Langmuir 2018, 34, 12318. 30226386 10.1021/acs.langmuir.8b02686

[26] a) M. Shin , H.‐A. Lee , M. Lee , Y. Shin , J.‐J. Song , S.‐W. Kang , D.‐H. Nam , E. J. Jeon , M. Cho , M. Do , S. H. Park , M. S. Lee , J.‐H. Jang , S.‐W. Cho , K.‐S. Kim , H. Lee , Nat. Biomed. Eng. 2018, 2, 304; 30936449 10.1038/s41551-018-0227-9

[27] H. Lee , W. I. Kim , W. Youn , T. Park , S. Lee , T. S. Kim , J. F. Mano , I. S. Choi , Adv. Mater. 2018, 30, 1805091. 10.1002/adma.201805091 30302842

[28] D. Su , C. L. Teoh , S. Sahu , R. K. Das , Y.‐T. Chang , Biomaterials 2014, 35, 6078. 24794926 10.1016/j.biomaterials.2014.04.035

[29] C. Xu , H. Zou , Z. Zhao , Z. Zheng , R. T. Kwok , J. W. Lam , H. H. Sung , I. D. Williams , S. Chen , L. Zheng , Adv. NanoBiomed Res. 2021, 1, 2000080.

[30] a) E. D. Bartzoka , H. Lange , G. Poce , C. Crestini , ChemSusChem 2018, 11, 3975; 30204941 10.1002/cssc.201801546

[31] a) H. T. P. Anh , C.‐M. Huang , C.‐J. Huang , Sci. Rep. 2019, 9, 11562; 31399620 10.1038/s41598-019-47978-9 PMC6688990

[32] a) K. Bromma , A. Bannister , A. Kowalewski , L. Cicon , D. B. Chithrani , Cancer Nanotechnol. 2020, 11, 8; 32849921 10.1186/s12645-020-00064-6 PMC7437649

[33] T. Liu , W. Liu , M. Zhang , W. Yu , F. Gao , C. Li , S.‐B. Wang , J. Feng , X.‐Z. Zhang , ACS Nano 2018, 12, 12181. 30458111 10.1021/acsnano.8b05860

[34] H. Maeda , J. Wu , T. Sawa , Y. Matsumura , K. Hori , J. Controlled Release 2000, 65, 271. 10.1016/s0168-3659(99)00248-5 10699287

[35] a) S. J. Lee , S. H. Park , J. F. Chung , W. Choi , H. K. Huh , Oncotarget 2017, 8, 58264; 28938553 10.18632/oncotarget.16811 PMC5601649

