# Supplementary material for: One‐Pot Synthesis of Customized Metal–Phenolic‐Network‐Coated AIE Dots for In Vivo Bioimaging
Source: Adv Sci (Weinh). 2022 Feb 8;9(11):2104997. doi: 10.1002/advs.202104997 (PMC9008423; doi:10.1002/advs.202104997)
Supplement: Supplementary file 1 — Supporting Information [file ADVS-9-2104997-s002.pdf]

ADVANCED SCIENCE
Open Access

Supporting Information

for *Adv. Sci.*, DOI 10.1002/advs.202104997

One-Pot Synthesis of Customized Metal–Phenolic-Network-Coated AIE Dots for In Vivo Bioimaging

Changhuo Xu, Chen Peng, Xueqin Yang, Ruoyao Zhang, Zheng Zhao*, Bo Yan, Jun Zhang, Junyi Gong, Xuewen He, Ryan T. K. Kwok, Jacky W. Y. Lam and Ben Zhong Tang**

ADVANCED SCIENCE
Open Access

Supporting Information

for *Adv. Sci.,* DOI: 10.1002/advs.202104997

One-Pot Synthesis of Customized Metal-Phenolic Network-Coated AIE Dots for *in Vivo* Bioimaging

Changhuo Xu, Chen Peng, Xueqin Yang, Ruoyao Zhang, Zheng Zhao*, Bo Yan, Jun Zhang, Junyi Gong, Xuewen He, Ryan T. K. Kwok, Jacky W. Y. Lam, and Ben Zhong Tang**

Supporting Information

One-Pot Synthesis of Customized Metal-Phenolic Network-Coated AIE Dots for *in Vivo* Bioimaging

Changhuo Xu, Chen Peng, Xueqin Yang, Ruoyao Zhang, Zheng Zhao*, Bo Yan, Jun Zhang, Junyi Gong, Xuewen He, Ryan T. K. Kwok, Jacky W. Y. Lam, and Ben Zhong Tang**

1. Materials and instruments

N-phenyl-4-(1,2,2-triphenylvinyl) aniline (TPEA) was synthesized according to the literature method.[1] All other chemicals and reagents were commercially available and used as received without further purification. Toluene was distilled via solvent distillation systems containing sodium as a drying agent and benzophenone as an indicator. ^{1}H and ^{13}C NMR spectra were measured on a Bruker AVII 400 NMR spectrometer using $CDCl_3$ as a deuterated solvent and tetramethylsilane (TMS) as an internal reference. High-resolution mass spectra (HRMS) were recorded on a GCT Premier CAB 048 mass spectrometer operating in MALDI-TOF mode. Single-crystal data collection was carried out on a Bruker Smart APEXII charge-coupled device (CCD) diffractometer using graphite monochromated Cu Kα radiation (λ = 1.54178 Å). The morphology of nanoparticles was observed using a transmission electron microscopy (TEM, JEM-2010F, 3JEOL, Japan) and an ultra-high resolution scanning electron microscope (Model: JSM-6700F and JSM-7100F). The size distribution was recorded by a Malvern Zetasizer Nano S. UV absorption spectra were measured on a Milton Roy Spectronic 3000 Array Spectrophotometer. Photoluminescence (PL) spectra were recorded on a Perkin-Elmer spectrofluorometer LS 55. The lifetime was measured on an Edinburgh FLSP 920 fluorescence spectrophotometer equipped with a xenon arc lamp (Xe900). Fluorescence quantum yields were measured using a Hamamatsu absolute PL quantum yield spectrometer C11347 Quantaurus-QY. The XPS spectra were recorded on a PHI 5600 (Physical Electronics) Multi-Technique system equipped with a 150 watt monochromatic Al Kα X-ray source (1486.6 eV). The content of Fe was determined by inductively coupled plasma mass spectrometry (ICP-MS, NexION 300X, PerkinElmer). Fluorescence images were captured on a

Nikon Eclipse 80i microscope. Laser confocal scanning microscopic images were acquired on a Zeiss laser scanning confocal microscope (LSM7 DUO) and analyzed using ZEN 2009 software (Carl Zeiss).

2. Synthesis of 2TPEA-AQ

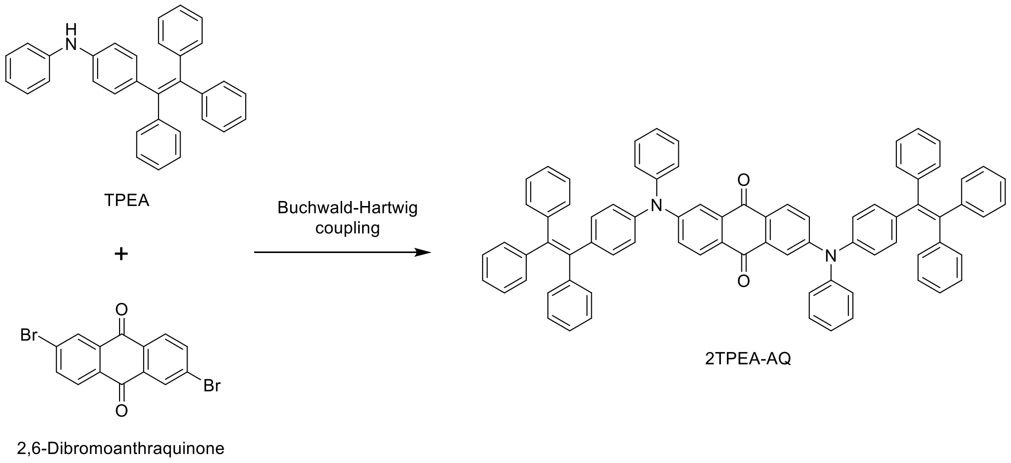

To a 100 mL two-necked round-bottom flask, TPEA (381 mg, 0.9 mmol), 2,6-Dibromoanthraquinone (110 mg, 0.3 mmol), Cs_2CO_3 (320 mg, 0.9 mmol), $Pd_2(dba)_3$ (28 mg, 0.03 mmol), and RuPhos (28 mg, 0.06 mmol) were added. The closed system was vacuumed and purged with dry nitrogen three times. Subsequently, 50 mL of dry toluene was injected. The reaction was stirred at room temperature and then heated to reflux for 24 h. After cooling down to room temperature, water was added to the mixture to terminate the reaction. The mixture was extracted with dichloromethane three times. The organic phase was combined and dried with anhydrous sodium sulfate. The solvent was removed under reduced pressure. The residue was purified with a silica gel chromatographic column using dichloromethane/hexane (*v*/*v* 1:1) as an eluent to afford 2ATPE-AQ as a red solid. Yield = 82%. ^{1}H NMR ($CDCl_3$, 400 MHz), δ (TMS, ppm): 8.05-8.07 (d, 2H, J = 8.8 Hz), 7.75-7.77 (d, 2H, J = 2.5 Hz), 7.33-7.39 (t, 4H, J = 7.8 Hz), 7.05-7.25 (m, 38H), 6.99-7.03 (d, 4H, J = 8.6 Hz), 6.89-

6.93 (d, 4H, J = 8.6 Hz). ^{13}C NMR ($CDCl_3$, 100 MHz), δ (TMS, ppm): 181.88, 152.83, 145.77, 144.06, 143.69, 143.50, 143.22, 141.43, 140.83, 140.26, 135.47, 132.71, 131.37, 131.32, 129.77, 128.92, 127.77, 127.73, 127.66, 126.66, 126.59, 126.49, 126.11, 126.08, 125.25, 125.20, 123. 52, 116.87. HRMS (MALDI-TOF), *m/z* calcd. for $C_{78}H_{54}N_2O_2$: 1050.4185; found 1050.4169 $[M]^+$.

3. Preparation of AIE-TM dots (M = Fe, Ti, Cu, or Ni)

To 4 mL of water under stirring, tannic acid (1.7 mg, 1 μmol) and metal ions (1 μmol) were added. Afterward, the AIEgen solution in THF (1 mg/mL, 1 mL) was dropwise added into the aforesaid aqueous solution. The pH of the suspension was subsequently raised by adding 1 mL of MOPS buffer (100 mM, pH 7.4). After dialysis against water, AIE-TM dots were centrifuged to remove the excess TA/M^{n+} complexes and stored at 4 ^oC for further use.

4. Preparation of DSPE-PEG-wrapped 2TPEA-AQ@AIE dots

To 4 mL of water under stirring, 1 mL of THF solution containing 1 mg of 2TPEA-AQ and 1 mg of DSPE-$mPEG_{2000}$ was dropwise added. After dialysis against water, the aqueous solution of AIE dots was obtained and stored at 4 ^oC for further use.

5. XPS characterization of 2TPEA-AQ@AIE-TM dots

The prepared aqueous solution of AIE-TM dots was placed onto a clean Si wafer, followed by the complete evaporation of water under vacuum. PHI 5600 Multi-Technique system with an Al Kα source was used for XPS characterization of the obtained samples. Background subtraction and curve fitting was performed on the XPS raw data by operating Gaussian-Lorentzian functions. C 1s peak was shifted to 285.0 eV for binding energy calibration.

6. Cell culture

A549 cells were grown in Dulbecco's Modified Eagle Medium (DMEM) supplemented with 10% fetal bovine serum (FBS), 1% penicillin and streptomycin in a 5% CO_2 incubator at 37 ^oC.

7. Cell imaging

Cells were grown and allowed to adhere for 48 h in a 35 mm petri dish with a

coverslip. The cell medium was replaced with fresh DMEM. The cells were firstly incubated with 1 μM of 2TPEA-AQ@AIE-TFe dots (1 μM of 2TPEA-AQ) for 5 h, followed by co-staining with 0.2 μM of LysoTracker Green for 15 min. After the coverslip was taken out and the dish was mounted, the stained cells were imaged by a laser scanning confocal microscope (LSM710, ZEISS). The excitation wavelength of AIE-TFe dots was 488 nm, and the wavelength range of the emission filter was 650-700 nm. The excitation wavelength of LysoTracker Green was 488 nm, and the wavelength range of the emission filter was 500-550 nm.

8. Cytotoxicity study

CCK8 assay was used to evaluate the cell viability. Briefly, A549 or L929 cells in 200 μL DMEM were seeded in a 96-well plate at a density of 1.0×10^4 cells per well. After 24-h incubation, the medium of each well was replaced with 200 μL fresh medium containing 1×PBS (control) and 2TPEA-AQ@AIE-TFe dots at different concentrations (0, 0.4, 0.6, 0.8, 1.2, 1.6, and 2.0 μM of 2TPEA-AQ, respectively) for 24 h. Afterward, 20 μL of CCK-8 was added into each well and A549 or L929 cells were incubated for another 4 h. The OD value of each well at 450 nm was recorded using the microplate reader. Mean and standard deviation (SD) of 6 parallels were reported for each sample.

9. Xenografted tumor models *in vivo*

All animal experiments were performed in accordance with the guidelines of the ethical committee of Shanghai Public Health Clinical Center and the regulations of the National Ministry of Health. Female BALB/c nude mice (15-20 g, 3-4 weeks old) were purchased from Shanghai Slac Laboratory Animal Center. 1×10^6 A549 cells were implanted subcutaneously into the left flank of each nude mouse. The tumor models were used for *in vivo* imaging when the volume of tumors reached 0.5-1 cm^3.

10. *In vivo* MR imaging and fluorescence imaging (intratumoral injection)

The tumor-bearing mice were anesthetized with pentobarbital sodium (40 mg kg^{-1} for each mouse) and treated with an intratumoral injection of 2TPEA-AQ@AIE-TFe dots ([2TPEA-AQ] = 100 μM, [Fe^{3+}] = 81 μM, 25 μL) for *in vivo* MR imaging and fluorescence imaging. As a control, the tumor-bearing mice were also administered with gadodiamide (Brand name: Omniscan, [Gd] = 81 μM, 25 μL) for MR imaging.

MR imaging was performed on a 3.0 T MR system (MAGNETOM VERIO, SIEMENS Medical Systems, Erlangen, Germany) with a custom-built animal receiver coil (Chenguang Med. Tech., Shanghai, China). T_1-weighted MR images were obtained at different time points (0, 10, 30, 60, 120 min) with the same setting parameters. MR signal to noise ratios (SNR) of tumors were defined as the signal intensity of the tumor region divided by the noise intensity of air around the tumor region. Time-course fluorescence images were acquired by using a Bruker In-vivo F PRO imaging system (Billerica, MA, USA). The excitation wavelength was 485 nm, and the collected emission wavelength range was 600-700 nm.

11. Histological examination

To study *in vivo* toxicity, the tumor-bearing mice were euthanized post the intratumoral injection of 2TPEA-AQ@AIE-TFe dots at different time points (12, 24, and 48 h). The tumors were extracted, sectioned, and stained with hematoxylin and eosin. The images of tumor sections were obtained using a Leica DM IL LED inverted phase contrast microscope.

12. *In vivo* MR imaging and fluorescence imaging (intravenous injection)

The tumor-bearing mice were intravenously injected with 2TPEA-AQ@AIE-TFe dots for *in vivo* MR imaging and fluorescence imaging. The same experimental protocol as intratumoral injection was adopted for MR imaging. Besides, after the intravenous injection of 2TPEA-AQ@AIE-TFe dots, the main organs including the heart, liver, spleen, lung, kidney, and tumor extracted at different time points (30 and 60 min) were sectioned. The images of histological sections were captured through a Leica DM6 B inverted fluorescence microscope.

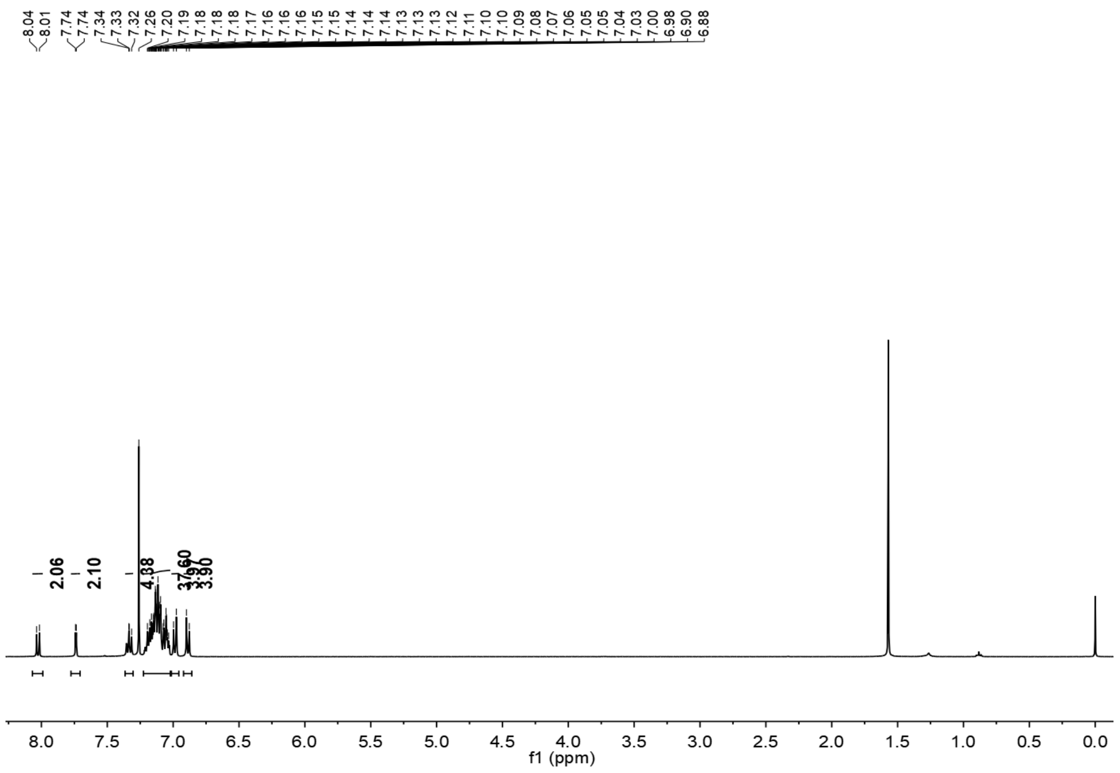

Figure S1. ^{1}H NMR spectrum of 2TPEA-AQ in $CDCl_3$.

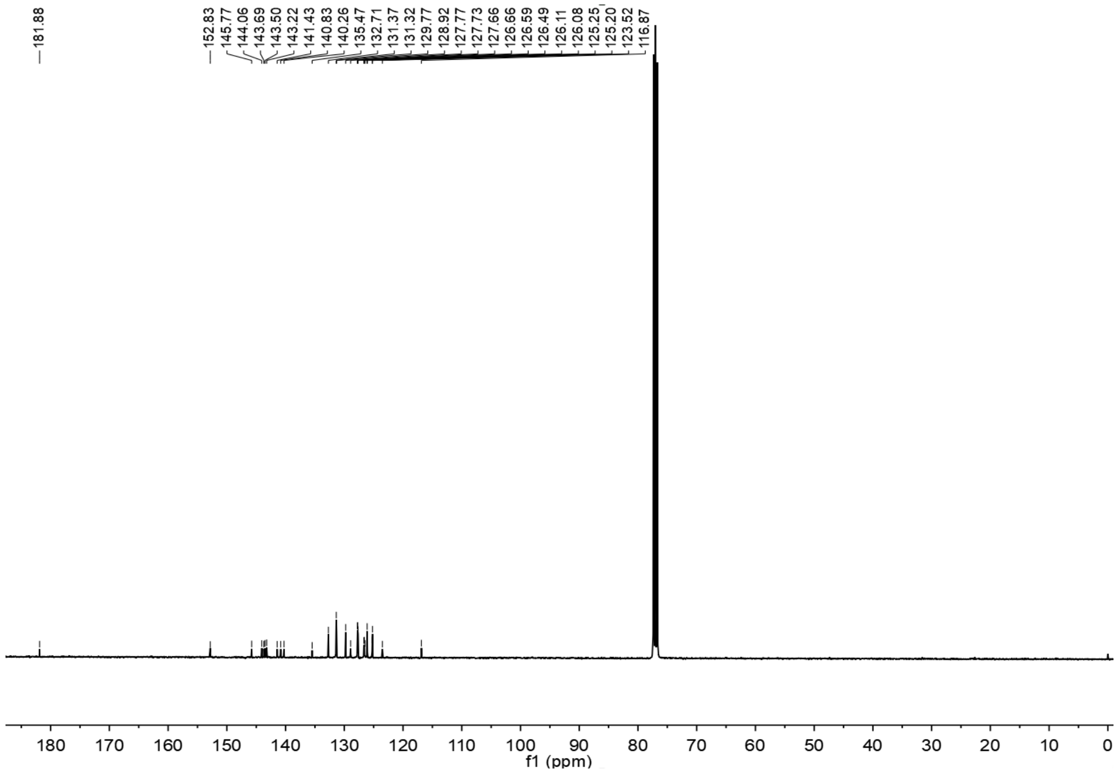

Figure S2. ^{13}C NMR spectrum of 2TPEA-AQ in $CDCl_3$.

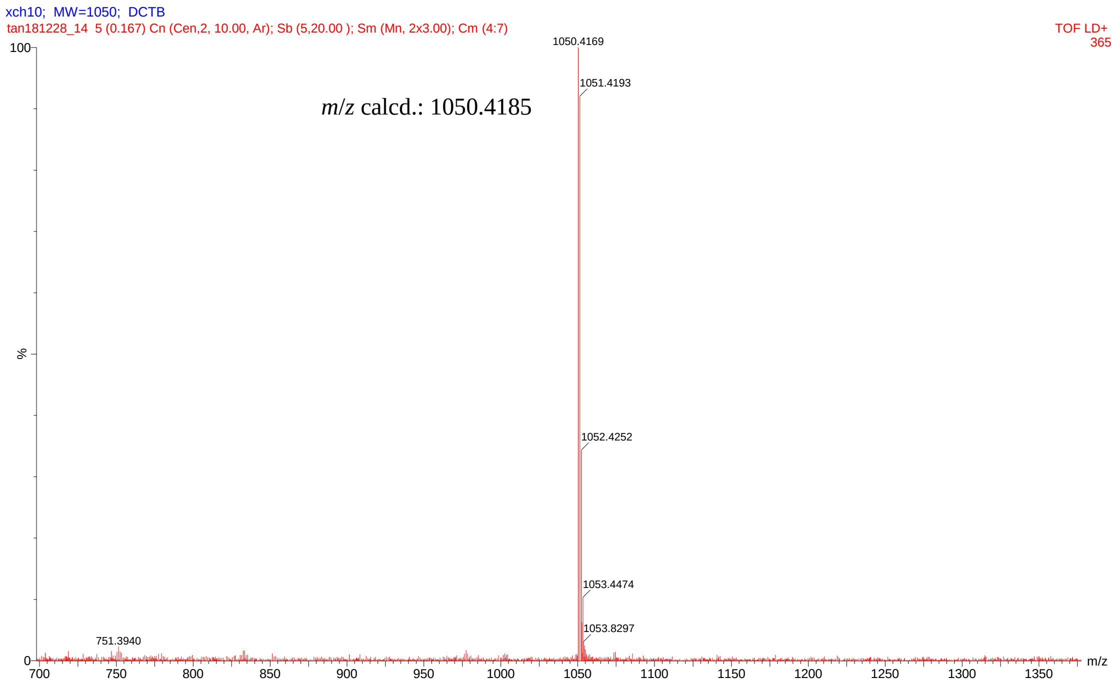

Figure S3. HRMS spectrum of 2TPEA-AQ.

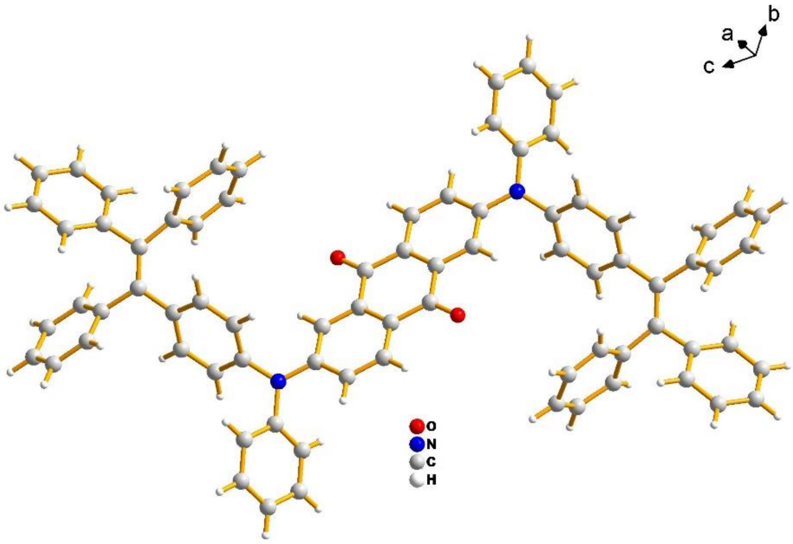

Table S1. Crystal data and structure refinement for 2TPEA-AQ.

Identification code	2TPEA-AQ	
Empirical formula	$C_{78}\ H_{54.4}\ N_2\ O_{2.2}$	
Formula weight	1054.83	
Temperature	100.15 K	
Wavelength	1.54184 Å	
Crystal system	Triclinic	
Space group	*P*-1	
Unit cell dimensions	a = 10.9200(5) Å	a= 105.952(4)°
	b = 11.4109(5) Å	b= 90.738(3)°
	c = 11.7518(5) Å	g = 102.523(4)°
Volume	1370.37(11) $Å^3$	
Z	1	
Density (calculated)	1.278 mg/m^3	
Absorption coefficient	0.588 mm^{-1}	
F(000)	554	
Crystal size	0.15 × 0.07 × 0.02 mm^3	
Theta range for data collection	3.924 to 62.499°.	
Index ranges	-10⩽h⩽12, -13⩽k⩽10, -13⩽l⩽13	
Reflections collected	6973	
Independent reflections	4330 [R(int) = 0.0143]	
Completeness to theta = 62.499°	99.0 %	
Absorption correction	Semi-empirical from equivalents	
Max. and min. transmission	1.00000 and 0.95183	
Refinement method	Full-matrix least-squares on F^2	
Data / restraints / parameters	4330 / 716 / 470	
Goodness-of-fit on F^2	1.138	
Final R indices [I>2σ(I)]	R_1 = 0.0589, wR_2 = 0.2308	
R indices (all data)	R_1 = 0.0623, wR_2 = 0.2401	
Largest diff. peak and hole	0.745 and -0.352 e. $Å^{-3}$	

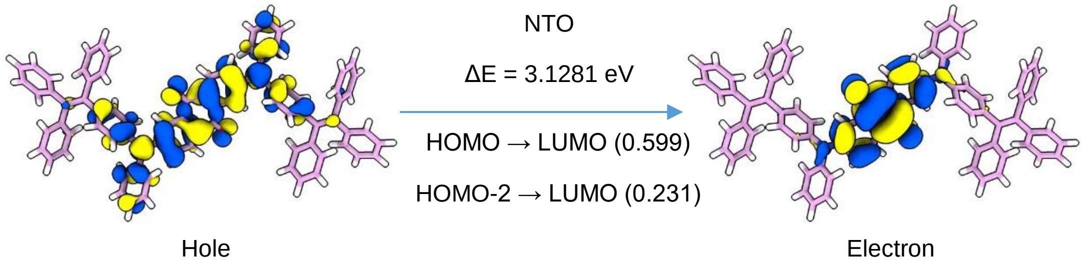

Figure S4. Natural transition orbital (NTO) analysis of 2TPEA-AQ optimized in the ground state .

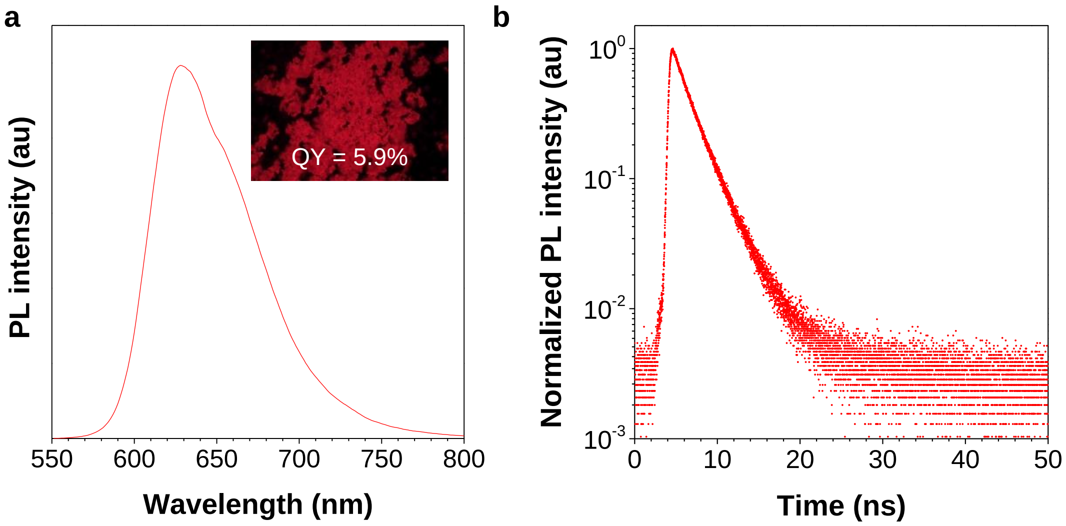

Figure S5. (a) PL spectrum of 2TPEA-AQ in the solid state. Insets: fluorescence image of 2TPEA-AQ in the solid state. (b) PL decay spectrum of 2TPEA-AQ in the solid state recorded at 300 K.

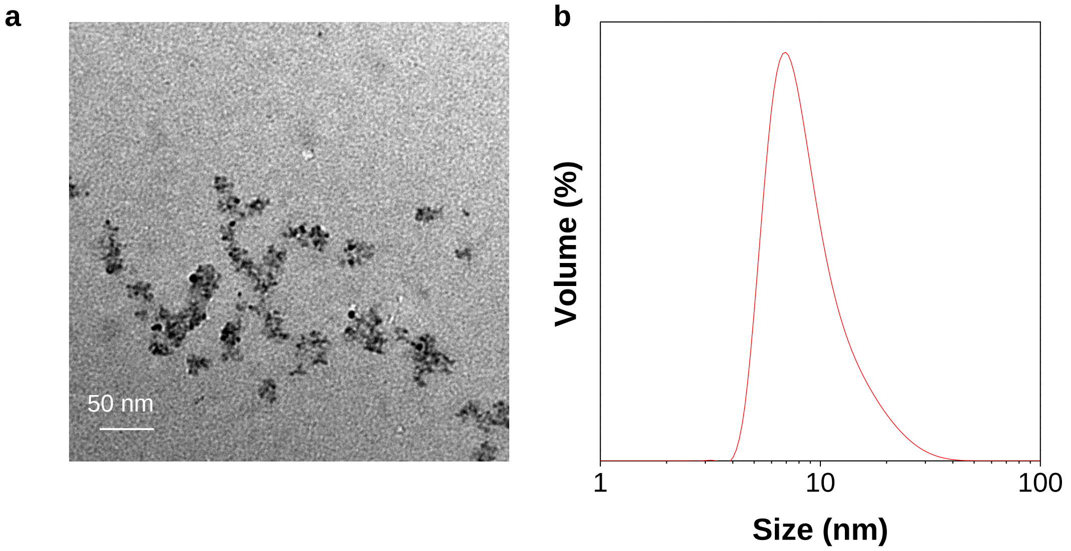

Figure S6. (a) TEM image and (b) the size distribution of TA/Fe^{3+} complexes formed in water. [Fe^{3+}] = [TA] = 0.2 mM.

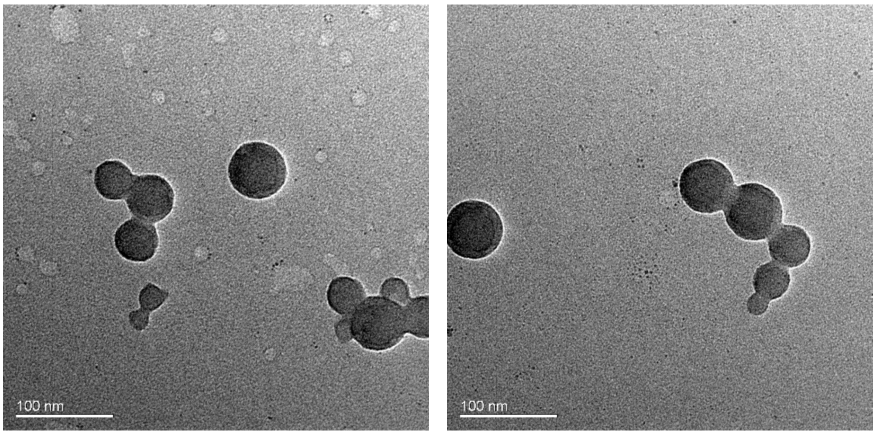

Figure S7. Additional TEM images of 2TPEA-AQ@AIE-TFe dots.

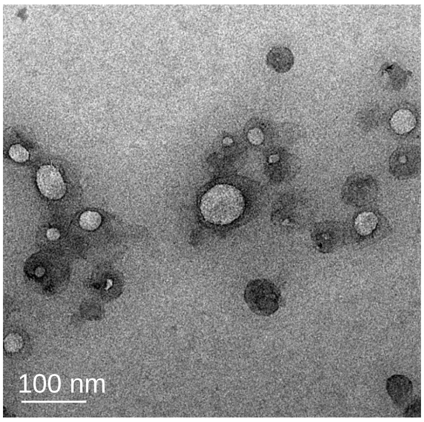

Figure S8. TEM image of THF-etched 2TPEA-AQ@AIE-TFe dots.

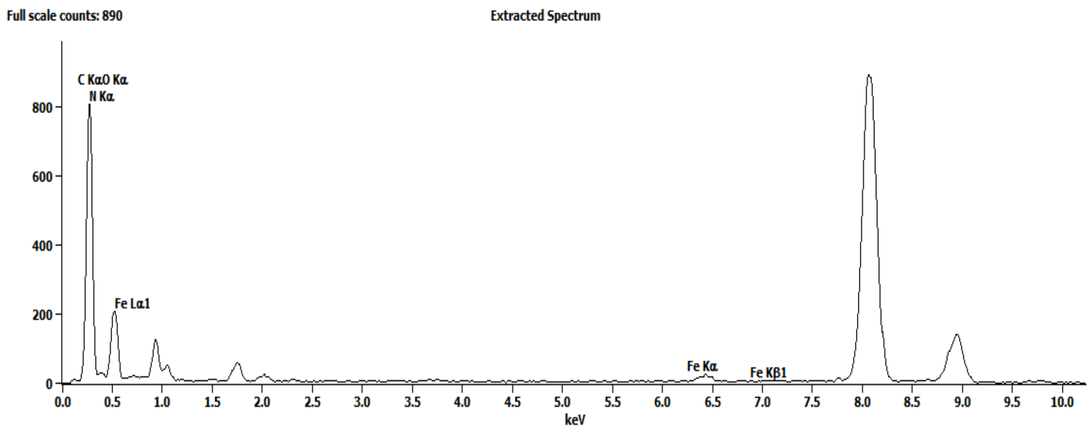

Figure S9. Energy-dispersive X-ray spectrum of 2TPEA-AQ@AIE-TFe dots.

Table S2. Quantitative results of elemental composition (C, N, O, and Fe) for the extracted spectrum.

Element Line	Net Counts	Int. Cps/nA	Weight %	Weight % Error	Norm. Wt.%	Norm. Wt.% Error	Atom %	Atom % Error
C K	5049	51.985	84.58	± 1.34	84.58	± 1.34	88.15	± 1.40
N K	361	3.717	3.81	± 0.72	3.81	± 0.72	3.41	± 0.64
O K	1778	18.307	10.47	± 0.48	10.47	± 0.48	8.19	± 0.37
Fe K	273	2.811	1.14	± 0.13	1.14	± 0.13	0.26	± 0.03
Total			100.00		100.00		100.00	

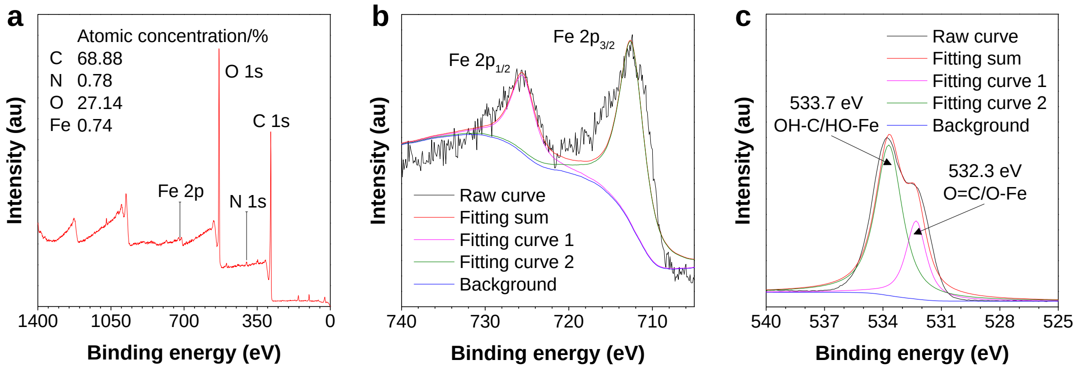

Figure S10. (a) XPS survey, (b) Fe 2p core-level, and (c) O 1s core-level spectra of 2TPEA-AQ@AIE TFe dots. Deconvoluted spectra are also shown.

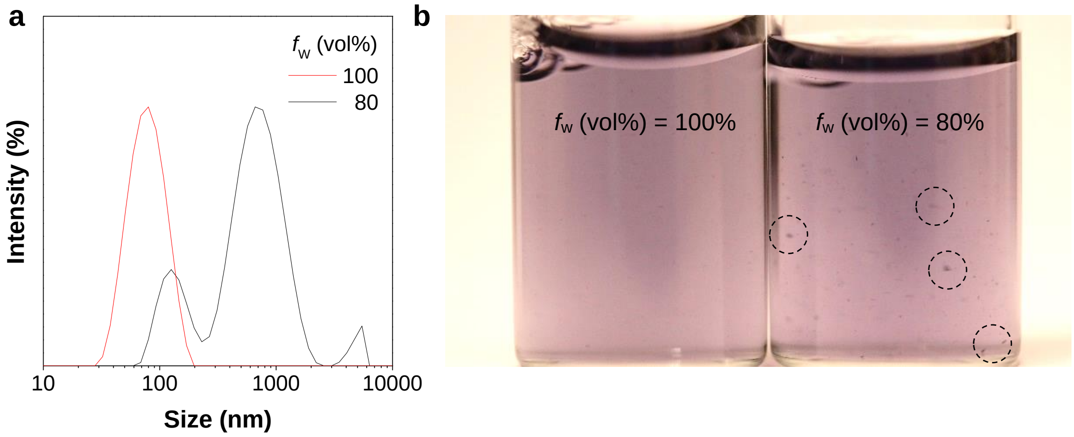

Figure S11. (a) Size distributions and (b) photos of TA/Fe^{3+} complexes formed in pure water or a THF/water mixture with a water fraction of 80% after standing for 12 h. [Fe^{3+}] = [TA] = 0.2 mM.

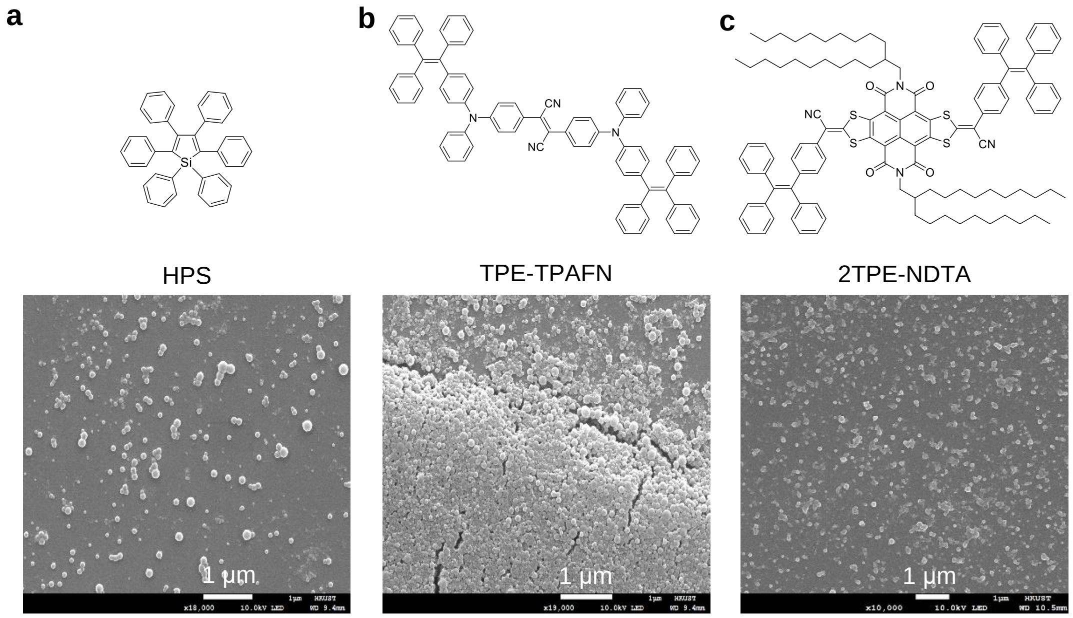

Figure S12. Chemical structures and SEM images of AIE-TFe dots fabricated by HPS, TPE-TPAFN, and 2TPE-NDTA. [AIEgen] = [Fe^{3+}] = [TA] = 0.2 mM.

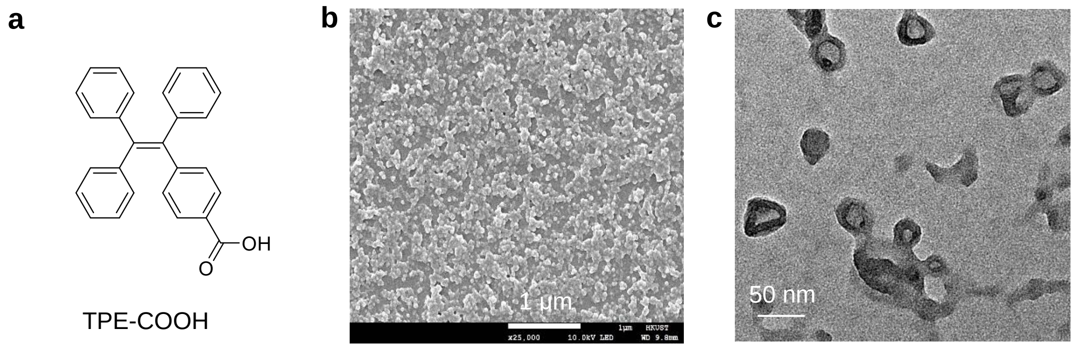

Figure S13. (a) Chemical structure of TPE-COOH. (b and c) (b) SEM and (c) TEM images of TPE-COOH@AIE-TFe vesicles. [TPE-COOH] = [Fe^{3+}] = [TA] = 0.2 mM.

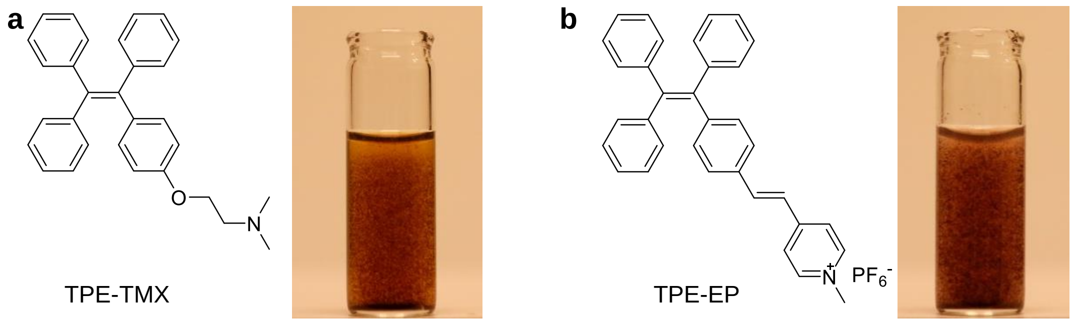

Figure S14. (a and b) Representative examples of positively charged AIEgens and the obtained solutions after the self-assembly of the positively charged AIEgens and TA/Fe^{3+} complexes. [AIEgen] = [Fe^{3+}] = [TA] = 0.2 mM.

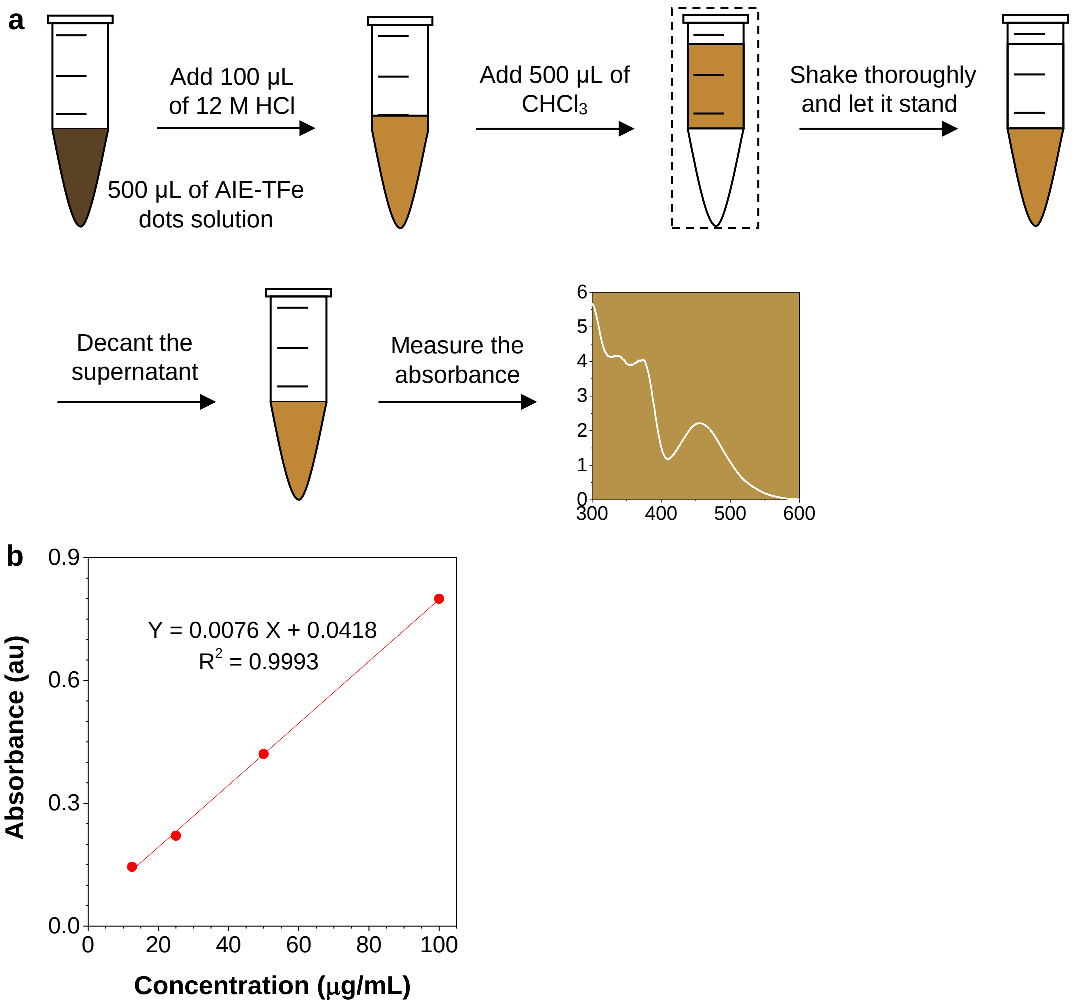

Figure S15. (a) Schematic illustration of UV-vis spectrophotometric method to determine the mass concentration of 2TPEA-AQ in 2TPEA-AQ@AIE-TFe dots. (b) Calibration curve of the absorbance of 2TPEA-AQ at 460 nm as a function of 2TPEA-AQ concentration in $CHCl_3$.

Table S3. Size, polydispersity index (PDI), zeta potential, and encapsulation efficiency (EE) of 2TPEA-AQ@AIE-TFe dots prepared at varying Fe^{3+} concentrations while the concentrations of 2TPEA-AQ and TA are fixed at 0.2 mM.

$[Fe^{3+}]$/mM	Size/nm	PDI	Zeta Potential/mV	EE/%
0.4	Precipitation			
0.2	95.46	0.115	-28.3	95
0.1	79.06	0.138	-32.2	98
0.05	106.19	0.192	-26.3	95
0.025	115.77	0.227	-22.4	96
0.0125	114.79	0.173	-23.6	80

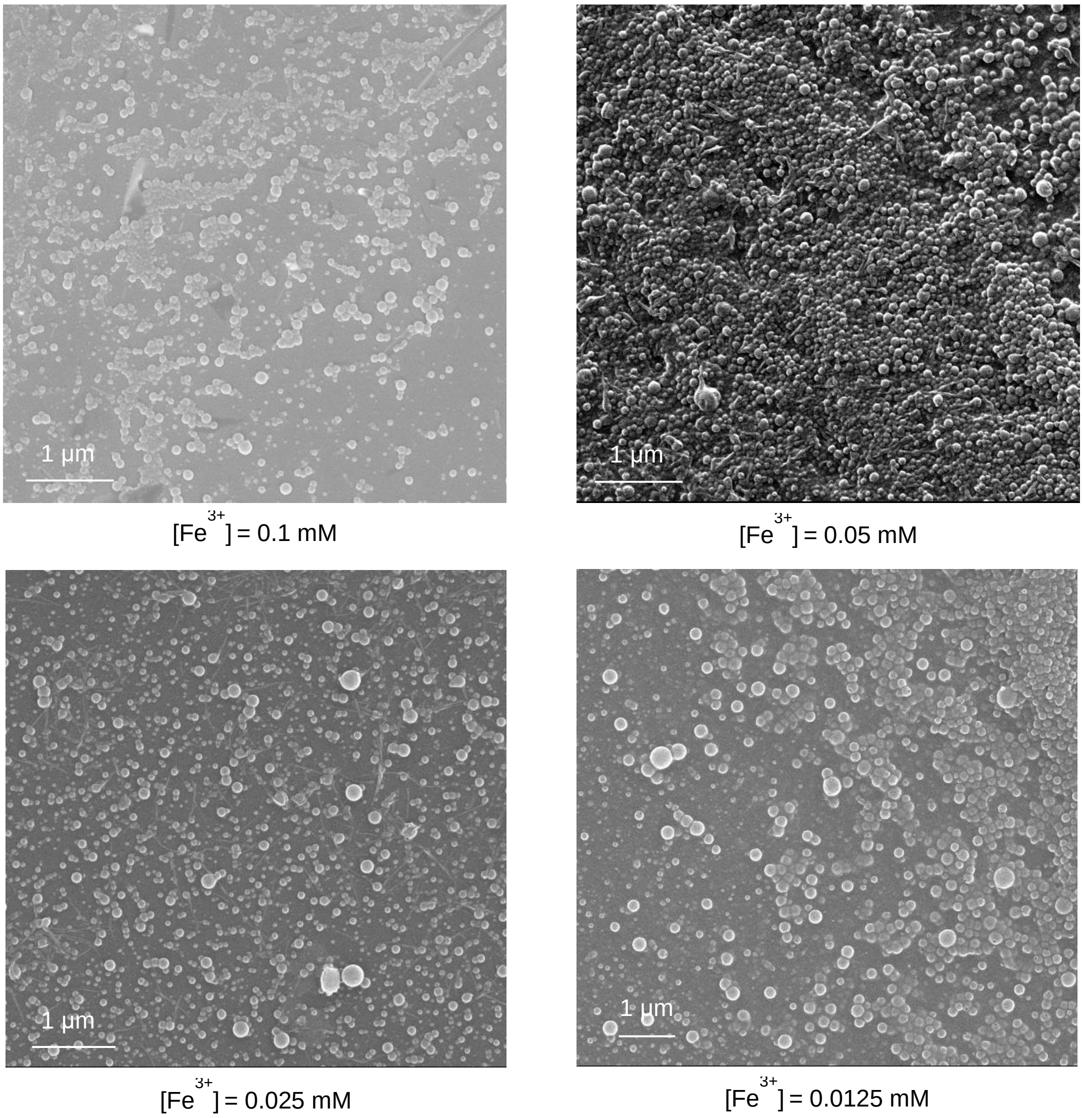

Figure S16. SEM images of 2TPEA-AQ@AIE-TFe dots fabricated at varying Fe^{3+} concentrations while the concentrations of 2TPEA-AQ and TA are fixed at 0.2 mM.

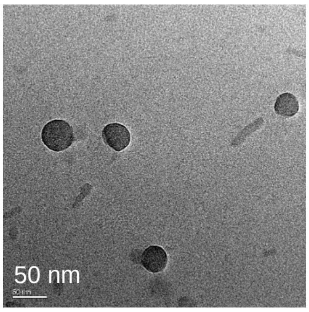

Figure S17. TEM image of DSPE-PEG-wrapping AIE dots fabricated by 2TPEA-AQ.

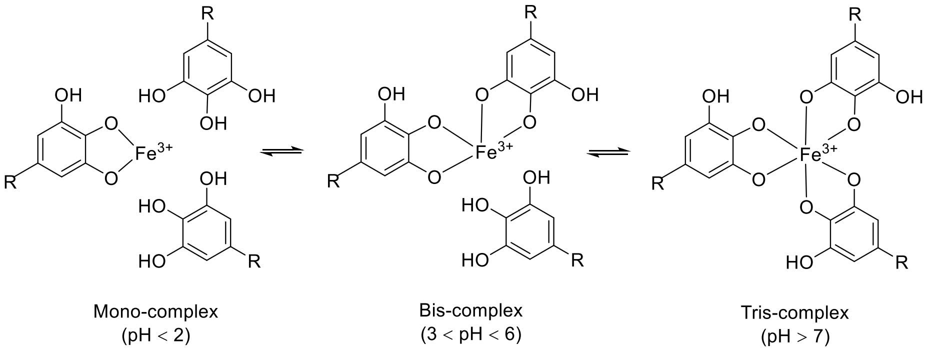

Scheme S1. pH-dependent transition of TA-Fe^{3+} complexation states. R represents the remainder of TA.[2]

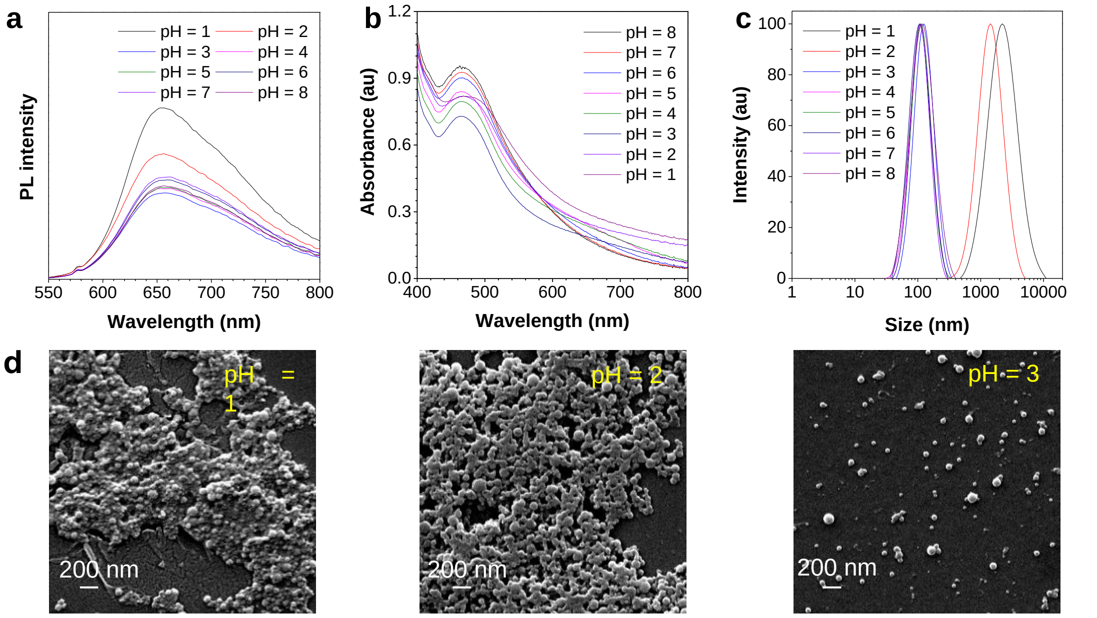

Figure S18. (a-c) (a) PL spectra, (b) absorption spectra, and (c) size distributions of 2TPEA-AQ@AIE-TFe dots in aqueous solutions at varying pH for 24 h. (d) SEM images of 2TPEA-AQ@AIE-TFe dots after incubated in aqueous solutions at pH = 1, pH = 2 and pH =3 for 24 h.

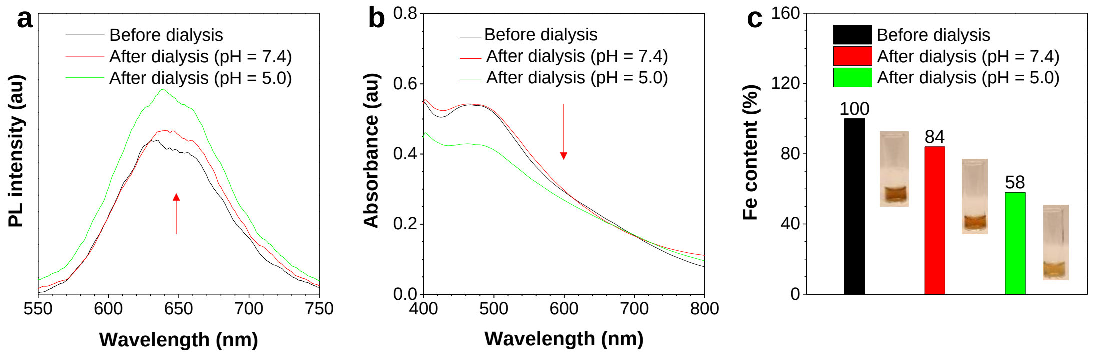

Figure S19. (a-c) (a) PL, (b) Absorbance, and (c) Fe content changes of 2TPEA-AQ@AIE-TFe dots after dialysis in phosphate buffer solutions at different pH (5.0 and 7.4) for 24 h. Insets: photos of nanoparticle solutions before and after dialysis in phosphate buffer solutions at different pH.

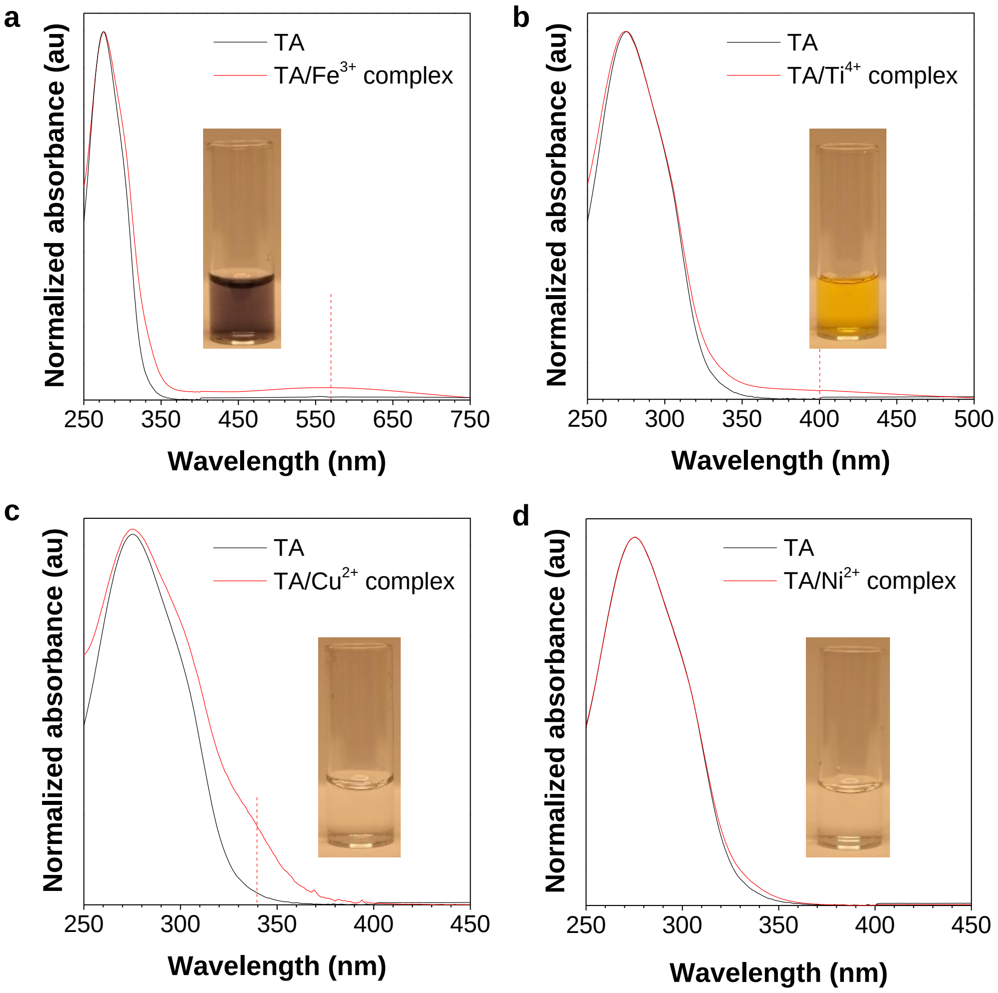

Figure S20. Absorption spectra of TA and TA/M^{n+} (M^{n+} = Fe^{3+}, Ti^{4+}, Cu^{2+}, or Ni^{2+}) complexes in deionized water. Insets: photos of different TA/M^{n+} complex solutions; [TA]:[M^{n+}] = 1:1.

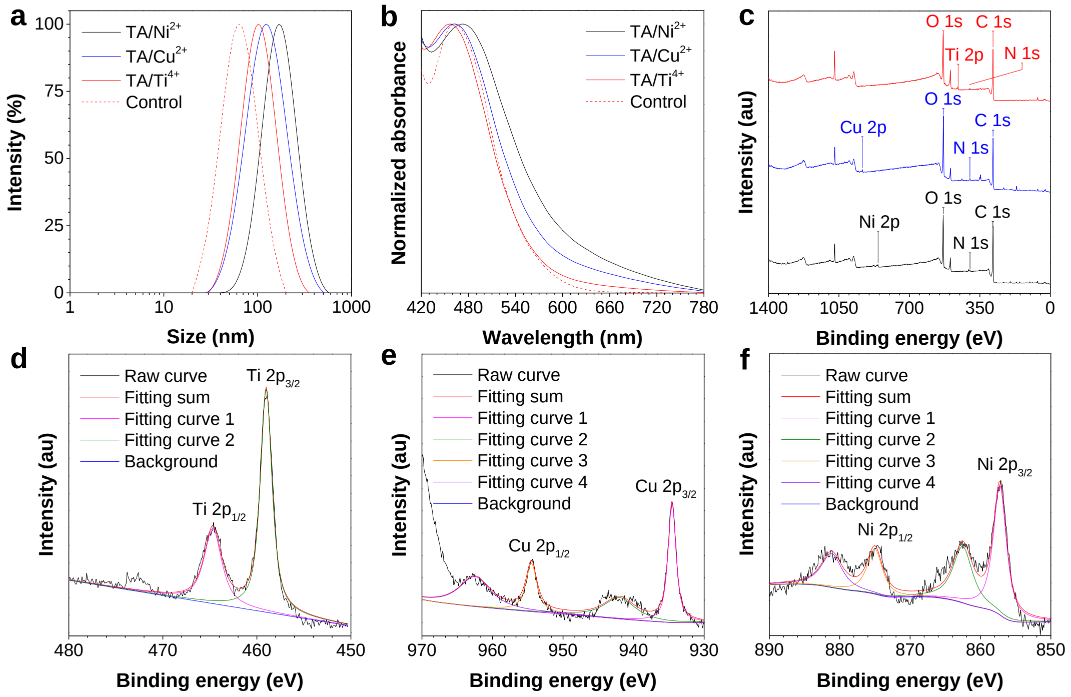

Figure S21. (a and b) (a) Size distributions, (b) absorption spectra, and (c) full-scale XPS spectra of 2TPEA-AQ@AIE-TM (M = Ti, Cu, or Ni) dots prepared by using different metal ions (M^{n+} = Ti^{4+}, Cu^{2+}, or Ni^{2+}). 2TPEA-AQ@AIE dots fabricated using DSPE-PEG as a surfactant were used as a control group; the concentration of 2TPEA-AQ in all the prepared AIE dots were 0.2 mM. (d-f) XPS metal 2p spectra with deconvoluted peaks of 2TPEA-AQ@AIE-TM (M = Ti, Cu, or Ni) dots.

Table S4. Quantification report of the major elemental composition of 2TPEA-AQ@AIE-TX (X = Ti, Cu, or Ni) dots measured by XPS analysis.

2TPEA-AQ@AIE-TTi dots		2TPEA-AQ@AIE-TCu dots		2TPEA-AQ@AIE-TNi dots	
Peak	Atomic con. /%	Peak	Atomic con. /%	Peak	Atomic con. /%
C 1s	70.86	C 1s	64.15	C 1s	71.82
N 1s	0.85	N 1s	2.32	N 1s	2.10
O 1s	21.04	O 1s	24.88	O 1s	19.83
Ti 2p	0.78	Cu 2p	0.34	Ni 2p	0.73

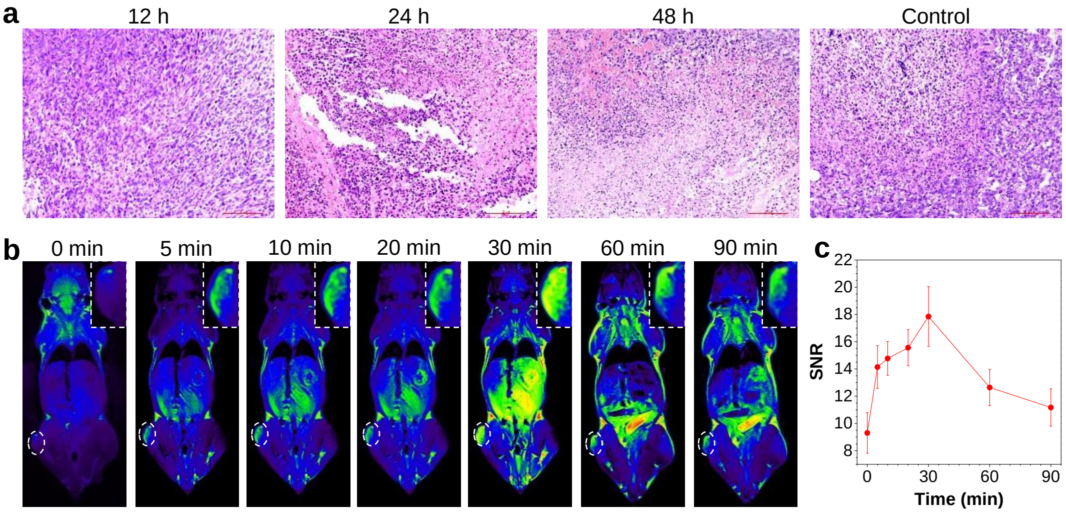

Figure S22. (a) H&E staining images of tumor sections at different time points after the mice were intratumorally injected with 2TPEA-AQ@AIE-TFe dots. Scale bar = 100 μm. (b) *In vivo* T_1-weighted MR pseudo-color images of A549 tumor-bearing mice following the intravenous injection of 2TPEA-AQ@AIE-TFe dots over a period of 90 min. The insets marked with the dashed line represent the tumor region. (c) MR SNR change in the tumor region with time after the intravenous injection of 2TPEA-AQ@AIE-TFe dots.

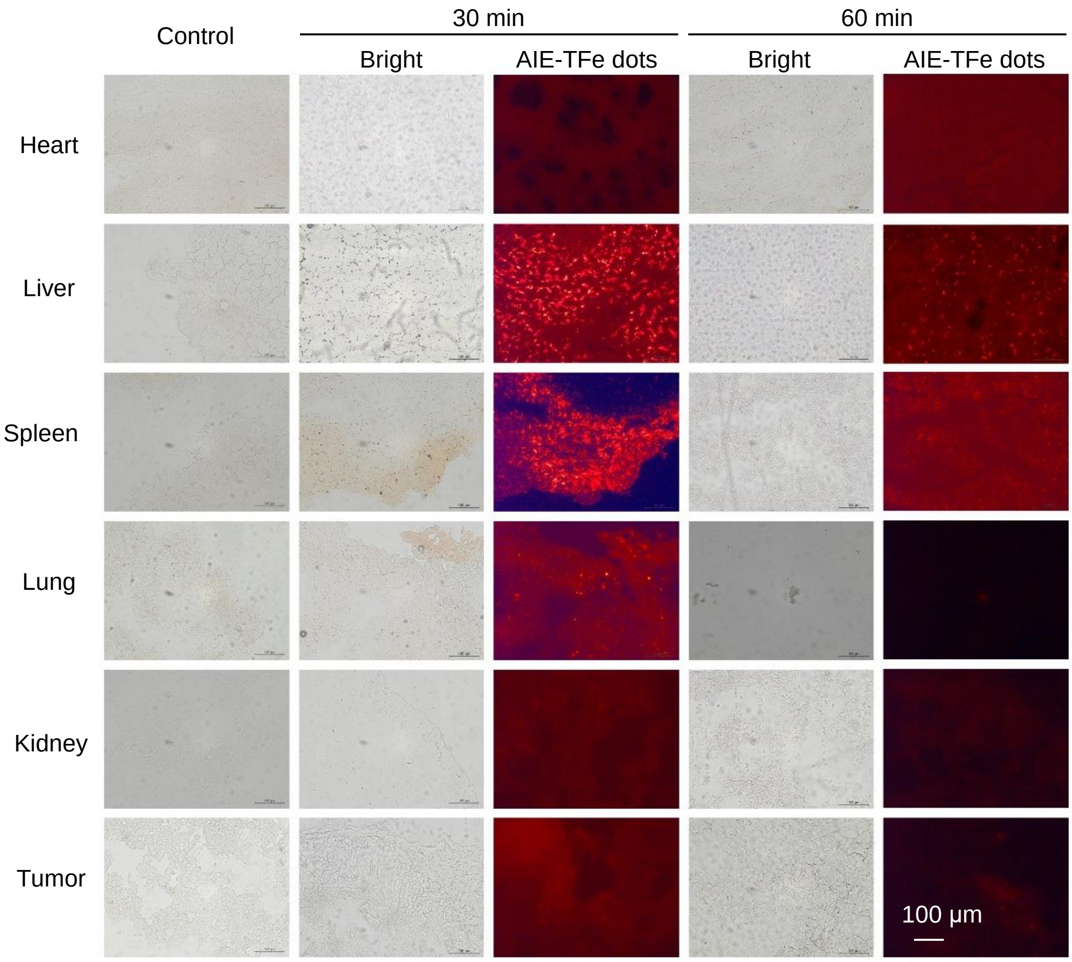

Figure S23. Representative *ex vivo* fluorescence images and photomicrographs of tissue sections harvested from the sacrificed mouse at different time points post intravenous injection of 2TPEA-AQ@AIE-TFe dots.

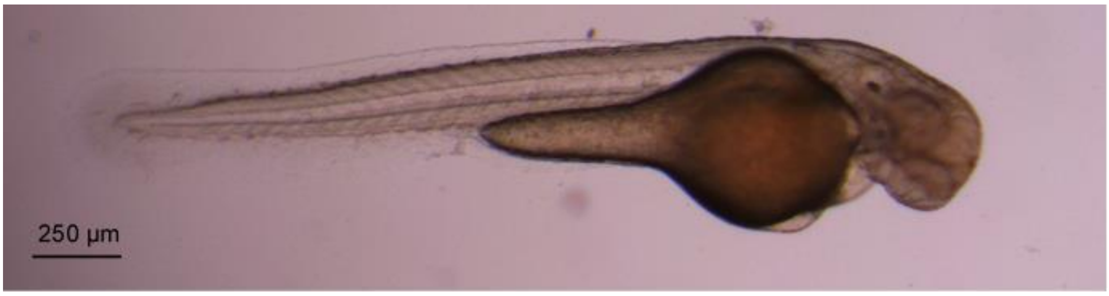

Figure S24. Photograph of a wild-type zebrafish larva (zebrafish strain: AB) at 2 days post-fertilization (dpf)

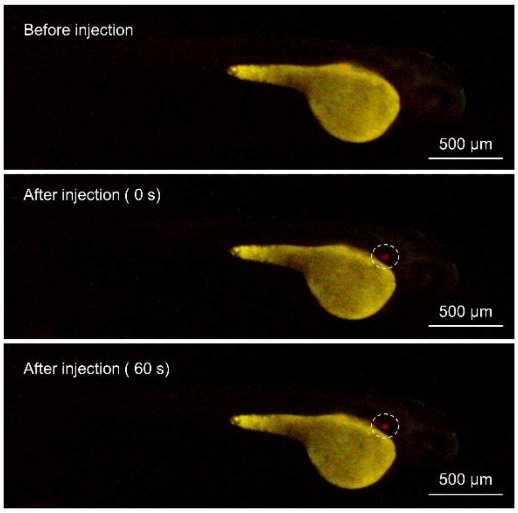

Figure S25. Fluorescent images of a wild-type zebrafish larva (zebrafish strain: AB) at different time points after injection of 2TPEA-AQ nanoaggregates into its heart. Excitation wavelength: 485 nm. The dashed line of ellipse indicates the injection region.

References

1. Lee, W. W.; Zhao, Z.; Cai, Y.; Xu, Z.; Yu, Y.; Xiong, Y.; Kwok, R. T.; Chen, Y.; Leung, N. L.; Ma, D., Facile access to deep red/near-infrared emissive AIEgens for efficient non-doped OLEDs. *Chemical science* **2018,** *9* (28), 6118-6125.
2. Ejima, H.; Richardson, J. J.; Liang, K.; Best, J. P.; van Koeverden, M. P.; Such, G. K.; Cui, J.; Caruso, F., One-step assembly of coordination complexes for versatile film and particle engineering. *Science* **2013,** *341* (6142), 154-157.
